# TGF-*β* inhibition can overcome cancer primary resistance to PD-1 blockade: A mathematical model

**DOI:** 10.1371/journal.pone.0252620

**Published:** 2021-06-01

**Authors:** Nourridine Siewe, Avner Friedman

**Affiliations:** 1 School of Mathematical Sciences, College of Science, Rochester Institute of Technology, Rochester, New York, United States of America; 2 Department of Mathematics, Mathematical Biosciences Institute, The Ohio State University, Columbus, Ohio, United States of America; University of Pécs Medical School, HUNGARY

## Abstract

Immune checkpoint inhibitors have demonstrated, over the recent years, impressive clinical response in cancer patients, but some patients do not respond at all to checkpoint blockade, exhibiting primary resistance. Primary resistance to PD-1 blockade is reported to occur under conditions of immunosuppressive tumor environment, a condition caused by myeloid derived suppressor cells (MDSCs), and by T cells exclusion, due to increased level of T regulatory cells (Tregs). Since TGF-*β* activates Tregs, TGF-*β* inhibitor may overcome primary resistance to anti-PD-1. Indeed, recent mice experiments show that combining anti-PD-1 with anti-TGF-*β* yields significant therapeutic improvements compared to anti-TGF-*β* alone. The present paper introduces two cancer-specific parameters and, correspondingly, develops a mathematical model which explains how primary resistance to PD-1 blockade occurs, in terms of the two cancer-specific parameters, and how, in combination with anti-TGF-*β*, anti-PD-1 provides significant benefits. The model is represented by a system of partial differential equations and the simulations are in agreement with the recent mice experiments. In some cancer patients, treatment with anti-PD-1 results in rapid progression of the disease, known as hyperprogression disease (HPD). The mathematical model can also explain how this situation arises, and it predicts that HPD may be reversed by combining anti-TGF-*β* to anti-PD-1. The model is used to demonstrate how the two cancer-specific parameters may serve as biomarkers in predicting the efficacy of combination therapy with PD-1 and TGF-*β* inhibitors.

## 1 Introduction

Immune checkpoint inhibitors, introduced in recent years, have demonstrated impressive clinical response in cancer patients, although resistance may develop over time. But some patients do not respond at all to checkpoint blockade, exhibiting, what is called, primary resistance. Mechanisms of adaptive resistance to PD-1 blockade and potential therapies to overcome it are reviewed in [1–5], and of primary resistance in [3–5]. In particular, primary resistance is reported to occur under conditions of immunosuppressive tumor environment, including effective T cells exclusion [4, 5]. Such environment is often caused by increased level of T regulatory cells (Tregs). Indeed, as reported in [6, 7], PD-1 expression balance between effective T cells and Tregs predicts the efficacy of PD-1 blockade therapy. In clinical study of patients with melanoma, PD-1 blockade resulting in decline of PD1^+^ Tregs predicted more favorable outcome [8]

In some cancer patients, treatment with anti-PD-1 resulted in rapid progression of tumor, known as hyperprogression disease (HPD) [8–10]. Recent reviews of HPD in cancer patients appeared in [11–13], and, of biomarkers for HPD, in [14]. Although the mechanism of HPD is unknown, it has been noted that HPD is associated with increased levels of MDSC and Treg cells [11, 14]. Motivated by the observation that HPD occurs in approximately 10% of anti-PD-1 monoclonal anti-body (mAb)-treated advanced gastric cancer patients, Kamada et al. [15] conducted mice experiments with gastric cancer. They demonstrated that PD-1 blockade activated and expanded tumor infiltration of PD-1^+^ Tregs to overwhelm tumor PD-1^+^ effective T cells, as cancer underwent rapid progression.

TGF-*β* is a pleiotropic cytokine that could suppress immune response by regulating Tregs [16]. Hence TGF-*β* blockade is likely to enhance immune-checkpoint therapy [17]. Mariathasan et al. [18] and Tauriello et al. [19] identified TGF-*β* signaling in tumor microenvironment as a determinant of tumor T cell role in affecting poor response to PD-1/PD-L1 blockade. They demonstrated, in mouse models, that combining TGF-*β* inhibition with immune checkpoint blockade induces complete and durable response to otherwise unresponsive tumor; see also reveiw article [20]. Sow et al. [21] found that combined inhibition of TGF-*β* signaling and PD-L1 is differentially effective in mouse model.

Streel et al. [22] and Martin et al. [23] have recently demonstrated, in several mouse models, that TGF-*β* inhibition overcomes primary resistance to PD-1 blockade. More precisely, in some cancers, PD-1 inhibition does not decrease tumor volume, but, in combination with anti-TGF-*β*, PD-1 blockade significantly improves outcome of treatment compared to treatment with anti-TGF-*β* alone. In this paper, we develop a mathematical model that explains these experimental results in [15, 22, 23] in terms of two cancer-specific parameters that may serve as cancer biomarkers.

The model is based on two important observations:

(i) TGF-*β* (*T*_*β*_) inhibits the killing rate of cancer cells by CD8^+^ T cells [24]; we represent this inhibition by a factor $1/(1+\zeta_{T_{\beta }}T_{\beta })$, for some constant $\zeta_{T_{\beta }}$.
(ii) The complex *Q* = PD-1/PD-L1 induces change from pro-inflammatory CD4^+^ T cells (*T*_1_) to regulatory T cells (*T*_*r*_) [25, 26], at rate modeled by $\lambda_{T_{1}T_{r}}T_{1}Q/(K_{Q}+Q)$, where *K*_*Q*_ and $\lambda_{T_{1}T_{r}}$ are constants.

Anti-PD-1 increases the activation of CD8^+^ T cells (*T*_8_). On the other hand, *T*_*β*_ contributes to the proliferation of *T*_*r*_ [27–28], possibly resulting in only minimal increase (*T*_8_).

PD-1 blockade increases the proliferation rate of *T*_1_. If *T*_1_ were fixed, the loss rate $\lambda_{T_{1}T_{r}}T_{1}Q/(K_{Q}+Q)$ of *T*_1_ (to *T*_*r*_) will also decrease. But since the proliferation of *T*_1_ has increased by the PD-1 blockade, the product *T*_1_ *Q*/(*K*_*Q*_+*Q*) may conceivably increase; in this case the rate of change $\lambda_{T_{1}T_{r}}T_{1}Q/(K_{Q}+Q)$ from *T*_1_ to *T*_*r*_ will increase, and, if $\lambda_{T_{1}T_{r}}$ is sufficiently large, the *T*_*r*_ inhibition of *T*_8_ may result in loss of *T*_8_, and thus in hyperprogression of cancer.

Myeloid cells play an important immunosuppressive role in the tumor microenvironment. They include MDSCs, M2 macrophages and M2-like TAMs (tumor associated macrophages) [29]. MDSCs secrete IL-10 [30, 31] and TGF-*β* [28, 32, 33]; M2 macrophages secrete IL-10 [34, 35], and TAMs and M2 macrophages secrete TGF-*β* [36]. For simplicity we shall represent these three types of myeloid populations by one variable, designated by *M*_2_, and will refer to it as MDSC or M2.

The mathematical model is represented by a system of partial differential equations within the tumor compartment. The species in the model include immune cells, CD8^+^ and CD4^+^-Th1 T cells, Tregs, immunosuppressive M2 macrophages and pro-inflammatory macrophages M1, and dendritic cells. They also include cytokines that play important role in the interactions among immune cells and cancer cells: CCL2 (MCP-1) and interleukins IL-2, IL-10 and IL-12. CCL2 is produced by cancer cells [37], and it attracts MDSCs into the tumor compartment [38–40]. IL-2 is produced by Th1 cells [41] and it enhances the proliferation of *T*_1_ and *T*_8_, but also *T*_*r*_, so its effect in clinical trials is not always predictable [42]. IL-12 is produced by dendritic cells and it activates *T*_1_ and *T*_8_ cells [43]. IL-10 is produced by MDSCs, M2 macrophages and cancer cells [30, 31]. Both IL-10 and *T*_*r*_ inhibit the activation of *T*_1_ and *T*_8_ by IL-12 [31]. The cancer-specific parameters $\lambda_{T_{1}T_{r}}$ and $\zeta_{T_{\beta }}$ play a critical role in the model simulations, and are adjusted in order to establish agreement with the experimental results of Streel et al. [22], Martin et al. [23], and Kamada et al. [15]. The model is then used to demonstrate how various other choices of these two parameters determine the efficacy of combination therapy with anti-PD-1 and anti-TGF-*β*, and how these parameters may serve as prediction biomarkers.

## 2 Mathematical model

**Fig 1:**
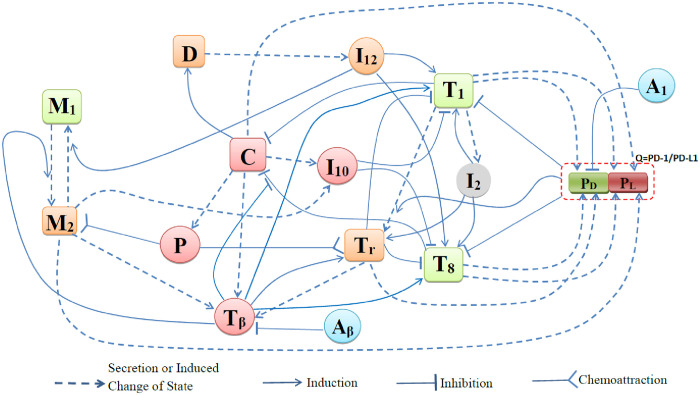
Network describing the interactions between cells and cytokines under treatment with anti-PD-1 and anti-TGF-*β*.

The mathematical model is based on the network shown in Fig 1. Table 1 lists the variables of the model in units of g/cm^3^. We assume that all species *X*_*i*_, (*i* = 1, …, *n*) are dispersing (or diffusing) with a coefficient $\delta_{X_{i}}$, and are dying (or degrading) at rate $\mu_{X_{i}}$; cells also undergo advection velocity **u** that is associated with internal pressure in the tumor compartment, see S1 File. We write the equation for cells *X*_*i*_ in the form

$$\begin{array}{c} \frac{\partial X_{i}}{\partial t}+\nabla \cdot \left(uX_{i}\right)-\delta_{X_{i}}\nabla^{2}X_{i}=F_{X_{i}}\left(X_{1},\ldots ,X_{n}\right) \end{array}$$

where ∇^2^ is the Laplace operator ∇⋅grad, or $\sum_{j=1}^{3}\frac{\partial^{2}}{\partial x_{j}^{2}}$. In modeling the structure of $F_{X_{i}}$ we use, for simplicity, the linear mas conservation law, that is, if *X*_*j*_+*X*_*k*_ → *X*_*m*_ then the rate by which *X*_*m*_ is formed, or *X*_*j*_ is lost, is *mX*_*j*_ *X*_*k*_ where *m* is a positive parameter. In a process where *X*_*i*_ is activated by cytokine *X*_*j*_, *X*_*j*_ represents molecules that are bound and internalized by *X*_*i*_, and this internalization may be limited owing to the limited rate of receptor recycling. We then represent the rate of activation by the Michaelis-Menten law *mX*_*i*_(*X*_*j*_/(*K*+*X*_*j*_)) for some positive parameters *m*, *K*. A term of the form *mX*_*i*_/(1+*X*_*j*_/*K*) means that *X*_*j*_ inhibits the growth of *X*_*i*_. Finally, an expression of the form ∇⋅(*X*_*i*_ *χ*∇*X*_*j*_) means that *X*_*i*_ is moving by chemotaxis in the direction of the gradient of chemoattractant *X*_*j*_ with chemotactic force *χ*, where *χ* is a positive parameter.

**Table 1:** Variables of the model. All concentrations are in units of g/cm^3^.

Variables	Descriptions	Variables	Descriptions
*M*_1_	density of M1 macrophages	*M*_2_	density of MDSCs
*D*	density of dedritic cells	*T*_1_	density of CD4^+^ T/Th1 cells
*T*_8_	density of CD8^+^ T cells	*T*_*r*_	density of Treg cells
*C*	density of cancer cells		
*I*_2_	concentration of IL-2	*I*_10_	concentration of IL-10
*I*_12_	concentration of IL-12	*P*	concentration of CCL2 (MCP-1)
*T*_*β*_	concentration of TGF-*β*	*P*_*D*_	concentration of PD-1
*P*_*L*_	concentration of PD-L1	*Q*	concentration of PD-1/PD-L1
*A*_1_	concentration of anti-PD-1	*A*_*β*_	concentration of anti-TGF-*β*

### 2.1 Equation for tumor cells (*C*)

We assume a logistic growth for cancer cells with carrying capacity *C*_*M*_, to account for space competition among these cells. Cancer cells are killed by CD8^+^ T cells, a processed inhibited by the pleiotropic cytokine TGF-*β* [24], represented by the factor $\frac{1}{1+\zeta_{T_{\beta }}T_{\beta }}$. We write the equation for *C* in the following form:

$$\begin{array}{c} \frac{\partial C}{\partial t}+\;\nabla \cdot \left(uC\right)-\delta_{C}\nabla^{2}C=\underset{\text{Growth}\;\text{of}\;\text{cancer}\;\text{cells}}{\underset{︸}{\lambda_{C}C(1-\frac{C}{C_{M}})}}-\underset{\text{killing}\;\text{by}\;T_{8}}{\underset{︸}{\frac{\mu_{T_{8}C}}{1+\zeta_{T_{\beta }}T_{\beta }}T_{8}C}}-\underset{\text{death}}{\underset{︸}{\mu_{C}C}}. \end{array}$$ (1)

### 2.2 Equation for M1 macrophages (*M*_1_)

The equation for M1 macrophages has the following form:

$$\begin{array}{cc} \frac{\partial M_{1}}{\partial t}+\nabla \cdot \left(uM_{1}\right)-\delta_{M}\nabla^{2}M_{1}= & \;\underset{\text{activation}\;\text{by}\;\text{CCL2}}{\underset{︸}{\lambda_{M_{1}}M_{0}\frac{P}{K_{P}+P}}}-\underset{\text{chemoattraction}\;\text{by}\;\text{CCL2}}{\underset{︸}{\nabla \cdot (\chi_{P}M_{1}\nabla P)}}\\ & \;+\underset{M_{2}\to M_{1}\;\text{by}\;\text{IL-12}}{\underset{︸}{\lambda_{M_{2}M_{1}}M_{2}\frac{I_{12}}{K_{I_{12}}+I_{12}}}}-\underset{M_{1}\to M_{2}\;\text{by}\;\text{TGF-}\beta }{\underset{︸}{\lambda_{M_{1}M_{2}}M_{1}\frac{T_{\beta }}{K_{T_{\beta }}+T_{\beta }}}}-\underset{\text{death}}{\underset{︸}{\mu_{M_{1}}M_{1}}} \end{array}$$ (2)

where the first term on the right-hand side represents a source of macrophages differentiated from monocytes that are activated by CCL2 (*P*) and the second term represents chemoattraction of *M*_1_ by CCL2 [44]. The third and fourth terms on the right-hand side represent phenotype changes from *M*_2_ to *M*_1_ induced by IL-12, and from *M*_1_ to *M*_2_ induced by TGF-*β* [44, 45].

### 2.3 Equation for MDSCs (*M*_2_)

Tumor recruits macrophages and “educates” them to become tumor-associated-macrophages (TAMs), which behave like MDSCs [46, 47]; MDSCs are chemotactically attracted by CCL2 [38–40]. The equation for *M*_2_ is given by:

$$\begin{array}{cc} \frac{\partial M_{2}}{\partial t}+\nabla \cdot \left(uM_{2}\right)-\delta_{M}\nabla^{2}M_{2}= & \;\underset{\text{activation}\;\text{by}\;\text{CCL2}}{\underset{︸}{\lambda_{M_{2}}M_{0}\frac{P}{K_{P}+P}}}-\underset{\text{chemoattratced}\;\text{by}\;\text{CCL2}}{\underset{︸}{\nabla \cdot (\chi_{P}M_{2}\nabla P)}}\\ & \;-\underset{M_{2}\to M_{1}\;\text{by}\;\text{IL-12}}{\underset{︸}{\lambda_{M_{2}M_{1}}M_{2}\frac{I_{12}}{K_{I_{12}}+I_{12}}}}+\underset{M_{1}\to M_{2}\;\text{by}\;\text{TGF-}\beta }{\underset{︸}{\lambda_{M_{1}M_{2}}M_{1}\frac{T_{\beta }}{K_{T_{\beta }}+T_{\beta }}}}-\underset{\text{death}}{\underset{︸}{\mu_{M_{2}}M_{2}}}. \end{array}$$ (3)

### 2.4 Equation for CD4^+^ T/Th1 cells (*T*_1_)

The pleiotropic cytokine TGF-*β* contributes to the development of naive CD4^+^ T cells, *T*_10_ [48]. Naive CD4^+^ T cells differentiate into Th1 cells under IL-12 inducement [41, 49], and this process is inhibited by IL-10 and Tregs. The proliferation of activated CD4^+^ T cells is enhanced by IL-2 [42]. Activation and proliferation of *T*_1_ cells are inhibited by the complex PD-1/PD-L1 (*Q*), represented by the factor $\frac{1}{1+Q/{\hat{K}}_{TQ}}$. The complex *Q* also mediates phenotype change from Th1 cells to Treg cells [25, 26], by a factor $\lambda_{T_{1}T_{r}}\frac{Q}{K_{Q}+Q}$; we consider the parameter $\lambda_{T_{1}T_{r}}$ to be cancer-specific. Hence *T*_1_ satisfies the following equation:

$$\begin{array}{cc} & \;\frac{\partial T_{1}}{\partial t}+\underset{\text{advection}}{\underset{︸}{\nabla \cdot (uT_{1})}}-\underset{\text{diffusion}}{\underset{︸}{\delta_{T}\nabla^{2}T_{1}}}=\\ & \;(\lambda_{T_{1}I_{12}}T_{10}\underset{T_{\beta }\text{-augmented}\;\text{activation}}{\underset{︸}{\left(1+\frac{T_{\beta }}{K_{T_{\beta }}+T_{\beta }}\right)}}\underset{\text{activation}\;\text{by}\;\text{IL-12}}{\underset{︸}{\frac{I_{12}}{K_{I_{12}}+I_{12}}}}\cdot \underset{\text{inhibition}\;\text{by}\;\text{IL-10}}{\underset{︸}{\frac{1}{1+I_{10}/{\hat{K}}_{TI_{10}}}}}\cdot \underset{\text{inhibition}\;\text{by}\;\text{Tregs}}{\underset{︸}{\frac{1}{1+T_{r}/{\hat{K}}_{TT_{r}}}}}+\\ & \;.\underset{\text{IL-2-induced}\;\text{proliferation}}{\underset{︸}{\lambda_{T_{1}I_{2}}T_{1}\frac{I_{2}}{K_{I_{2}}+I_{2}}}})\times \underset{\text{inhibition}\;\text{by}\;Q}{\underset{︸}{\frac{1}{1+Q/{\hat{K}}_{TQ}}}}-\underset{Q\text{-induced}\;T_{1}\to T_{r}\;\text{transition}}{\underset{︸}{\lambda_{T_{1}T_{r}}T_{1}\frac{Q}{K_{Q}+Q}}}-\underset{\text{death}}{\underset{︸}{\mu_{T_{1}}T_{1}}}. \end{array}$$ (4)

### 2.5 Equation for activated CD8^+^ T cells (*T*_8_)

The cytokine TGF-*β* contributes to the development of inactive CD8^+^ T cells, *T*_80_ [48]. Inactive CD8^+^ T cells are activated by IL-12 [41, 49], and this process is resisted by IL-10 and Treg cells [27, 31]. IL-2 enhances the proliferation of activated CD8^+^ T cells [42]. Both processes of activation and proliferation are inhibited by PD-1/PD-L1, by the factor $\frac{1}{1+Q/{\hat{K}}_{TQ}}$. Hence, *T*_8_ satisfies the following equation:

$$\begin{array}{cc} & \;\frac{\partial T_{8}}{\partial t}+\nabla \cdot \left(uT_{8}\right)-\delta_{T}\nabla^{2}T_{8}=\\ & \;(\lambda_{T_{8}I_{12}}T_{80}\underset{T_{\beta }\text{-augmented}\;\text{activation}}{\underset{︸}{\left(1+\frac{T_{\beta }}{K_{T_{\beta }}+T_{\beta }}\right)}}\underset{\text{activation}\;\text{by}\;\text{IL-12}}{\underset{︸}{\frac{I_{12}}{K_{I_{12}}+I_{12}}}}\cdot \underset{\text{inhibition}\;\text{by}\;\text{IL-10}}{\underset{︸}{\frac{1}{1+I_{10}/{\hat{K}}_{TI_{10}}}}}\cdot \underset{\text{inhibition}\;\text{by}\;\text{Tregs}}{\underset{︸}{\frac{1}{1+T_{r}/{\hat{K}}_{TT_{r}}}}}+\\ & \;.\underset{\text{IL-2-induced}\;\text{proliferation}}{\underset{︸}{\lambda_{T_{8}I_{2}}T_{8}\frac{I_{2}}{K_{I_{2}}+I_{2}}}})\times \underset{\text{inhibition}\;\text{by}\;Q}{\underset{︸}{\frac{1}{1+Q/{\hat{K}}_{TQ}}}}-\underset{\text{death}}{\underset{︸}{\mu_{T_{8}}T_{8}}}. \end{array}$$ (5)

### 2.6 Equation for Tregs (*T*_*r*_)

Naive CD4^+^ T cells differentiate into Tregs under activation by Fox3+ transcription factor, a process enhanced by TGF-*β* [27, 28]. The activated Tregs are recruited into tumor by tumor-derived immunosuppressive cytokines IL-6 and CCL2 (*P*) [38–40]; for simplicity, we represent both cytokines by CCL2. IL-2 enhances the proliferation of Tregs within the tumor [42] Representing this chemoattraction by ∇⋅(*χ*_*P*_ *T*_*r*_∇*P*), we get the following equation for *T*_*r*_:

$$\begin{array}{c} \frac{\partial T_{r}}{\partial t}+\;\nabla \cdot \left(uT_{r}\right)-\delta_{T}\nabla^{2}T_{r}=\\ \;\underset{T_{\beta }\text{-enhanced}\;\text{naive}\;\text{T}\;\text{cells}\;\text{activation}}{\underset{︸}{\lambda_{T_{r}T_{\beta }}T_{10}\frac{T_{\beta }}{K_{T_{\beta }}+T_{\beta }}}}+\underset{Q\text{-induced}\;T_{1}\to T_{r}\;\text{transition}}{\underset{︸}{\lambda_{T_{1}T_{r}}T_{1}\frac{Q}{K_{Q}+Q}}}+\;\underset{\text{IL-2-induced}\;\text{proliferation}}{\underset{︸}{\lambda_{T_{r}I_{2}T_{r}}\frac{I_{2}}{K_{I_{2}}+I_{2}}}}\\ \;-\underset{\text{chemoattraction}\;\text{by}\;\text{CCL2/MCP-1}}{\underset{︸}{\nabla \cdot (\chi_{P}T_{r}\nabla P)}}-\underset{\text{death}}{\underset{︸}{\mu_{T_{r}}T_{r}}}, \end{array}$$ (6)

where the second term in the right-hand side is the same as in Eq (4).

### 2.7 Equation for TGF-*β* (*T*_*β*_)

When anti-TGF-*β* drug is applied, TGF-*β* is depleted at a rate proportional to *A*_*β*_, and the equation for *T*_*β*_ takes the following form:

$$\begin{array}{cc} \frac{\partial T_{\beta }}{\partial t} & \;-\delta_{T_{\beta }}\nabla^{2}T_{\beta }=\underset{\text{secretion}\;\text{by}\;C\text{,}\;M_{2}\;\text{and}\;T_{r}}{\underset{︸}{\lambda_{T_{\beta }C}C+\lambda_{T_{\beta }M_{2}}M_{2}+\lambda_{T_{\beta }T_{r}}T_{r}}}-\underset{\text{degradation}}{\underset{︸}{\mu_{T_{\beta }}T_{\beta }}}-\underset{\text{depletion}\;\text{by}\;\text{anti-TGF-}\beta }{\underset{︸}{\mu_{A_{\beta }T_{\beta }}T_{\beta }A_{\beta }}}. \end{array}$$ (7)

The equations for *I*_2_, *I*_10_, *I*_12_, *P*, as well as the equations for *P*_*D*_ and *P*_*L*_ are given in S1 File, and we take

$$\begin{array}{c} Q=\sigma P_{D}P_{L}, \end{array}$$ (8)

for some parameter *σ*.

### 2.8 Equation for anti-PD-1 (*A*_1_)

In mice experiments in [23], anti-PD-1 was injected, intraperitoneally twice a week, begining *t*_0_ days after tumor cells implantation, and ending at day *t*_1_; in [22] the drug was administered daily. We approximate the effective source of the drug by taking it to be a constant, $\gamma_{A_{1}}$, so that

$$\begin{array}{c} c_{A_{1}}\left(t\right)=\{\begin{array}{cc} \gamma_{A_{1}}, & \text{if}\;t_{0}\leq t\leq t_{1}\\ 0, & \text{otherwise.} \end{array} \end{array}$$ (8)

The drug is depleted in the process of blocking PD-1, so that

$$\begin{array}{c} \frac{\partial A_{1}}{\partial t}-\delta_{A_{1}}\nabla^{2}A_{1}=\underset{\text{source}}{\underset{︸}{c_{A_{1}}\left(t\right)}}-\underset{\text{depletion}\;\text{through}\;\text{blocking}\;\text{PD-1}}{\underset{︸}{\mu_{P_{D}A_{1}}P_{D}A_{1}}}-\underset{\text{degradation}}{\underset{︸}{\mu_{A_{1}}A_{1}}} \end{array}$$ (9)

### 2.9 Equation for anti-TGF-*β* (*T*_*β*_)

In [22, 23], anti-TGF-*β* was administered weekly for the same periods *t*_0_ ≤ *t* ≤ *t*_1_ as in (8). We again approximate the effective level of the drug by taking

$$\begin{array}{c} c_{A_{\beta }}\left(t\right)=\{\begin{array}{cc} \gamma_{A_{\beta }}, & \text{if}\;t_{0}\leq t\leq t_{1}\\ 0, & \text{otherwise} \end{array} \end{array}$$ (10)

where $\gamma_{A_{\beta }}$ is some constant. The drug *A*_*β*_ is depleted in the process of blocking TGF-*β*, so that

$$\begin{array}{c} \frac{\partial A_{\beta }}{\partial t}-\delta_{A_{\beta }}\nabla^{2}A_{\beta }=\underset{\text{source}}{\underset{︸}{c_{A_{\beta }}\left(t\right)}}-\underset{\text{depletion}\;\text{through}\;\text{blocking}\;\text{TGF-}\beta }{\underset{︸}{\mu_{T_{\beta }A_{\beta }}T_{\beta }A_{\beta }}}-\underset{\text{degradation}}{\underset{︸}{\mu_{A_{\beta }}A_{\beta }}} \end{array}$$ (11)

#### 2.10 Equation for cells velocity (u)

The velocity **u** is determined by the condition that the combined density of all cells in the tumor compartment is constant; see S1 File.

To simplify the computations, we assume that the tumor is spherical and that all the densities and concentrations are radially symmetric, that is, functions of (*r*, *t*), 0 ≤ *r* ≤ *R*(*t*) where *r* = *R*(*t*) is the boundary of the tumor, and that **u** = *u*(*r*, *t*)**e**_*r*_, where **e**_*r*_ is the unit radial vector.

#### 2.11 Equation for free boundary (*R*)

We assume that the free boundary *r* = *R*(*t*) moves with the velocity of cells, so that

$$\begin{array}{c} \frac{dR(t)}{dt}=u\left(R\left(t\right),t\right). \end{array}$$ (12)

We complement the system by prescribing initial and boundary conditions; see S1 File.

## 3 Results

All the computations were done using Python 3.5.4. The parameter values of the model equations are estimated in and are listed in S1 File. The technique used in the simulations is also described in S1 File.

### 3.1 Mouse models and simulations

We define the efficacy of treatment by

$$\begin{array}{c} \text{efficacy}=\frac{\text{tumor}\;\text{volume}\;\text{with}\;\text{no}\;\text{treatment}-\text{tumor}\;\text{volume}\;\text{under}\;\text{treatment}}{\text{tumor}\;\text{volume}\;\text{with}\;\text{no}\;\text{treatment}}\times 100\%, \end{array}$$ (13)

where both volumes are measured at the last day of treatment. We refer to efficacy as the relative difference (in tumor volume) of treatment to no treatment, in percentage. Negative efficacy means that treatment resulted in increase (rather than decrease) in tumor volume.

**Fig 2:**
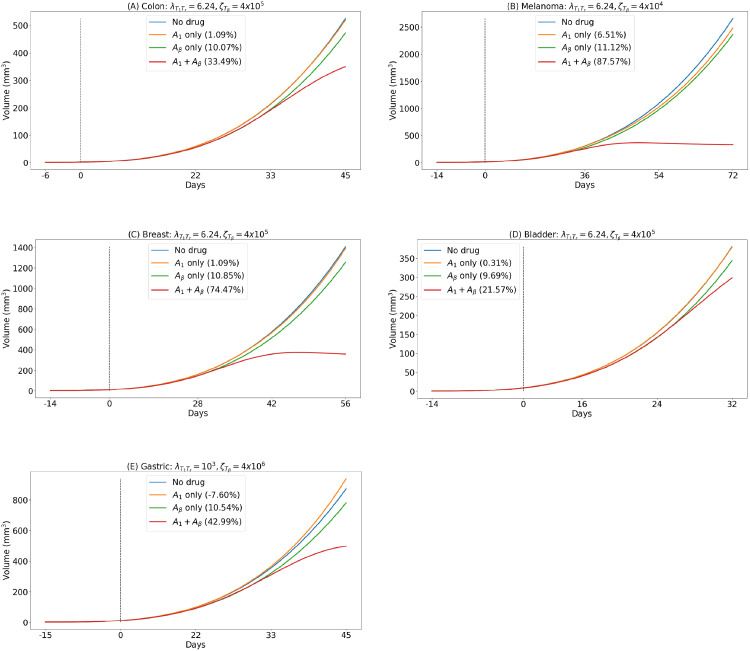
Tumor volume under various combinations with anti-PD-1 and anti-TGF-*β*. The “%” represents the difference, in volume, of treatment to no treatment, in percentage. All parameters are as in S1 File, with $\gamma_{A_{1}}=10^{-8}\;\text{g}/{\text{cm}}^{3}\cdot \text{d}$ and $\gamma_{A_{\beta }}=2\times 10^{-6}\;\text{g}/{\text{cm}}^{3}\cdot \text{d}$. (A) Colon cancer: treatment starts at day 6 which corresponds to the schedule in [22]. (B) Melanoma cancer: treatment starts at day 14 as in [22]. (C) Breast cancer: treatment starts at day 14 as in [23]. (D) Bladder cancer: treatment starts at day 14 which corresponds to the schedule in [23]. (E) Gastric cancer: treatment starts at day 15 which corresponds to the schedule in [15].

Streel et al. [22] and Martin et al. [23] performed mice experiments with different types of cancer, treated with combinations of anti-PD-1 and anti-TGF-*β*. In Streel et al. [22] (Fig. 2b), mice were implanted with colon cancer cells and treatment began 6 days after infection. The tumor volume in each mouse was measured regularly for 45 days and reported accordingly. They found that there was almost no reduction in the tumor size when treatment was with anti-PD-1 alone, but the tumor volume reduced significantly when anti-PD-1 was combined with anti-TGF-*β*. Our simulations in Fig 2A show the volume of the tumor in the cases of no treatment and treatment with various combinations of anti-PD-1 and anti-TGF-*β*. We see that while anti-PD-1 as a single agent does not reduce the cancer volume growth, when given in combination with anti-TGF-*β*, the growth of the tumor volume is significantly decreased; this is in agreement with Fig. 2b in [22]

In the experiments conducted by Martin et al. [23], mice were implanted with cells from bladder cancer, melanoma or breast cancer, and then treated with anti-PD-1 as a single agent, or with combination of anti-PD-1 and anti-TGF-*β*. Starting treatment at day 14 post-infection, Martin et al. found, as in [22], that in the case of breast cancer ([23] Fig 4B, 4C) and bladder cancer ([23] Fig 4G, 4H), with anti-PD-1 alone there was hardly any reduction in the tumor volume, but in combination with anti-TGF-*β*, anti-PD-1 reduced tumor volume significantly; Fig 2C and 2D are in agreement with these results. On the other hand, in the case of melanoma ([23] Fig 4D, 4E), there was primary resistance to anti-PD-1; Fig 2B is in agreement with this result. Note that the cancer-specific parameter $\zeta_{T_{\beta }}$ in Fig 2B is much smaller than the corresponding parameter in Fig 2A, 2D and 2E.

Note that the parameters $\lambda_{T_{1}T_{r}}$ and $\zeta_{T_{\beta }}$ are the same in Fig 2A, 2C and 2D, but the profiles are taken for different time durations (45, 56 and 32 days, respectively), and this accounts for the somewhat different impressions one may get of the tumor volume growth.

In mice experiments with gastric cancer, Kamada et al. [15] administered anti-PD-1 as a single agent and compared the tumor volume in this case to the tumor volume in the control (no-drug) case. They observed that the tumor volume with anti-PD-1 exceeded the tumor volume in the control case (Fig 6B, 6C in [15]). The simulations in Fig 2E show the same qualitative results. Notice that in these simulations, the parameter $\zeta_{T_{\beta }}$ is the same as in Fig 2A, 2C and 2D, but $\lambda_{T_{1}T_{r}}$ is much larger than in these figures.

Fig 2E shows also the effect of anti-PD-1 on tumor treated with anti-TGF-*β*: In the first few weeks, tumor volume slightly increases (hyperprogression of cancer) but later on it decreases, and by day 45 it is significantly decreased under the combined therapy.

### 3.2 Tumor volume hyperprogression

**Fig 3:**
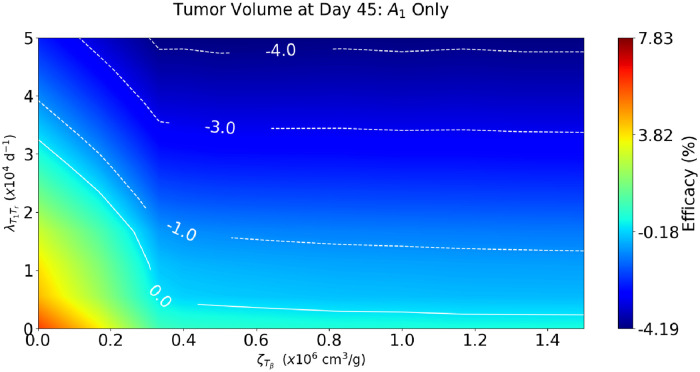
Combined effect of cancer-specific parameters $\lambda_{T_{1}T_{r}}$ and $\zeta_{T_{\beta }}$, under treatment with anti-PD-1, at $\gamma_{A_{1}}=10^{-8}\;\text{g}/{\text{cm}}^{3}\cdot \text{d}$. The color column indicates relative difference of the tumor volume at day 45. Negative values represent parameter ranges of tumor hyperprogression.

The simulations in Fig 2 suggest that hyperprogression of cancer under PD-1 inhibition depends on the parameters $\lambda_{T_{1}T_{r}}$ and $\zeta_{T_{\beta }}$. Fig 3 shows tumor volume at day 45 for pairs of parameters $\left(\zeta_{T_{\beta }},\lambda_{T_{1}T_{r}}\right)$ in the range $0<\zeta_{T_{\beta }}<1.5\times 10^{6}$ cm^3^/g, $0<\lambda_{T_{1}T_{r}}<5\times 10^{4}{\text{d}}^{-1}$. The color column scales the efficacy, that is, the percentage of increase/decrease of tumor volume relative to the control case; the drug level is taken to be $\gamma_{A_{1}}=10^{-8}\;\text{g}/{\text{cm}}^{3}\cdot \text{d}$. We see that (i) negative efficacy (hyperprogression) increases with both $\lambda_{T_{1}T_{r}}$ and $\zeta_{T_{\beta }}$, and (ii) efficacy is positive when either $\lambda_{T_{1}T_{r}}$ or $\zeta_{T_{\beta }}$ is small. A monotone decreasing curve of the form $\lambda_{T_{1}T_{r}}=f(\zeta_{T_{\beta }})$ separates the regions of positive and negative efficacies.

**Fig 4:**
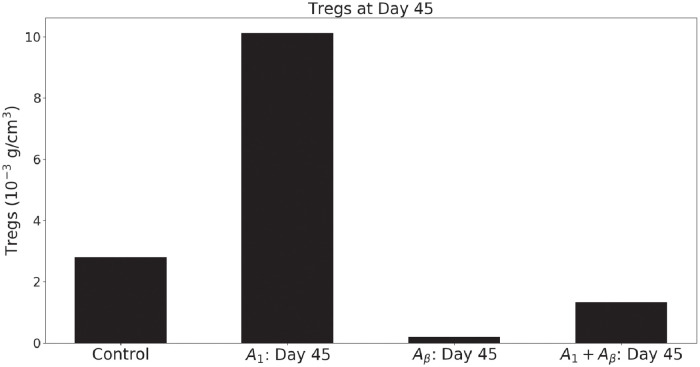
Tregs levels in all treatment combinations with anti-PD-1 and anti-TGF-*β*. The bar plots represent the density of Tregs in the control, anti-PD-1 only, anti-TGF-*β* only, and anti-PD-1+anti-TGF-*β* cases. Tregs increase with anti-PD-1 as single agent, decrease significantly with anti-TGF-*β* as single agent, and decrease (but remains below the control case) when anti-PD-1 is combined with anti-TGF-*β*.

**Fig 5:**
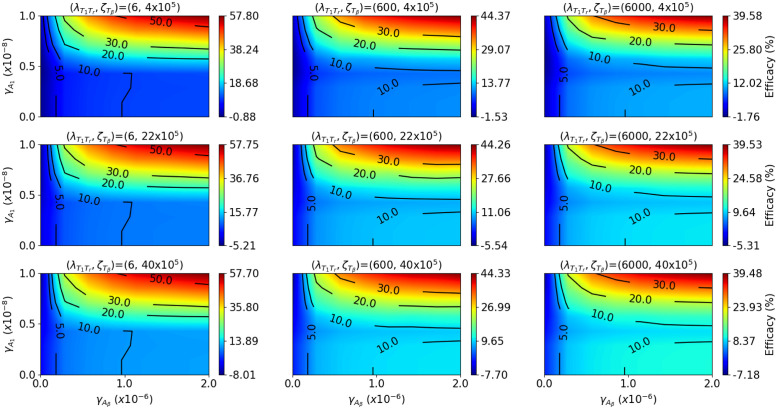
Efficacy map, combination of anti-PD-1 with anti-TGF-*β*. We vary the cancer-specific parameters $\lambda_{T_{1}T_{r}}\in \{6,6\times 10^{2},6\times 10^{3}\}{\text{d}}^{-1}$ and $\zeta_{T_{\beta }}\in \{4\times 10^{5},22\times 10^{5},40\times 10^{5}\}\;{\text{cm}}^{3}/\text{g}$, and plot efficacy maps for the combination anti-PD-1+anti-TGF-*β* with doses $\gamma_{A_{1}}$ between $0<\gamma_{A_{1}}<10^{-8}\;\text{g}/{\text{cm}}^{3}\cdot \text{d}$ and $\gamma_{A_{\beta }}$ between 0 < *γ*_*β*_ < 2×10^−6^ g/cm^3⋅^d, respectively. The color columns indicate the relative difference of the tumor at day 45. Negative values represent anti-PD-1 and anti-TGF-*β* dose ranges of tumor hyperprogression.

Kamada et al. [15] (Fig. 5F) also measured the level of Tregs under treatment with anti-PD-1 as single agent, and compared it with the corresponding level of Tregs in the control case. They found that Tregs level increased by 1/3 more than their corresponding level in the control case. The simulations in Fig 4 show the same level of increase of Tregs under treatment with anti-PD-1, with cancer-specific parameters $\zeta_{T_{\beta }}=4\times 10^{6}\;{\text{cm}}^{3}/\text{g}$ and $\lambda_{T_{1}T_{r}}=10^{3}\;{\text{d}}^{-1}$. Fig 4 also shows that the Tregs level is very low under treatment with anti-TGF-*β*, but it increases significantly (although it remains below the control case) in combination with anti-PD-1.

### 3.3 Efficacy maps

In order to see how the cancer-specific parameters affect the efficacy of treatment, we took 9 pairs $\left(\lambda_{T_{1}T_{r}},\zeta_{T_{\beta }}\right)$ as in Fig 5 and for each pair, we simulated the model under combination therapy with $\left(\gamma_{A_{\beta }},\gamma_{A_{1}}\right)$ that vary in the region $0<\gamma_{A_{1}}<10^{-8}\;\text{g}/{\text{cm}}^{3}\cdot \text{d}$ $0<\gamma_{A_{\beta }}<2\times 10^{-6}\;\text{g}/{\text{cm}}^{3}\cdot \text{d}$. Then, in Fig 5, we plotted the efficacy of treatment after 45 days. Note that the values of $\lambda_{T_{1}T_{r}}$ increase along each row, and the values of $\zeta_{T_{\beta }}$ increase along each column. The ranges of $\lambda_{T_{1}T_{r}}$ and $\zeta_{T_{\beta }}$, and the ranges of $\gamma_{A_{1}}$ and $\gamma_{A_{\beta }}$ include the values that appear in Fig 2.

From Fig 5 we see that (i) for any combination $\left(\gamma_{A_{\beta }},\gamma_{A_{1}}\right)$, the efficacy increases when $\lambda_{T_{1}T_{r}}$ and $\zeta_{T_{\beta }}$ are decreased. (ii) For large values of $\lambda_{T_{1}T_{r}}$ and $\zeta_{T_{\beta }}$, tumor progression is likely to occur. (iii) For small values of $\lambda_{T_{1}T_{r}}$ and $\zeta_{T_{\beta }}$, the efficacy increases as $\gamma_{A_{1}}$ and $\gamma_{A_{\beta }}$ are increased. We also see that efficacy always increases if $\gamma_{A_{\beta }}$ is increased. This is not surprising, since if $\gamma_{A_{\beta }}$ is increased, the *T*_*β*_ is decreased and, hence, the killing rate of *C* by *T*_8_, which is proportional to $1/(1+\zeta_{T_{\beta }}T_{\beta })$ is increased.

On the other hand, as seen in the last two columns of Fig 5, for fixed large $\gamma_{A_{\beta }}$, there is an interval $\left(\gamma_{A_{1}}^{-},\gamma_{A_{1}}^{+}\right)$ such that the efficacy is decreasing as $\gamma_{A_{1}}$ increases in this interval. To explain this situation we note that if $\gamma_{A_{1}}$ is increased then *T*_1_ and *T*_8_ are increased, but also *T*_*r*_ is increased, at rate $\lambda_{T_{1}T_{r}}T_{1}$, and hence *T*_*β*_ is also increased (by Eq (7)). It follows that the killing rate of *C*, which is proportional to $T_{8}/(1+\zeta_{T_{\beta }}T_{\beta })$, may either increase or decrease. Fig 5 shows that, as $\gamma_{A_{1}}$ increases, this factor decreases as long as $\gamma_{A_{1}}$ remains in an intermediate interval $\left(\gamma_{A_{1}}^{-},\gamma_{A_{1}}^{+}\right)$, and is increased elsewhere.

## 4 Conclusion

Therapeutic antibodies that block PD-1/PD-L1 induce robust and durable response in some cancer patients, negative response in some patients [12, 14], and no response at all in others [18, 50]. Since substantial proportion of patients have little or no benefits, while treatment with these drugs are costly and might have associated toxicity [51], biomarkers which are likely to predict response rate to PD-1/PD-L1 blockade are highly desirable [51, 52]. You et al. [53] summarizes (in Table 1) clinical outcome of predictive biomarkers for PD-1/PD-L1 blockade, while asserting the need for reliable biomarkers to ensure rational use of this checkpoint blockade. In the present paper we identified two cancer-specific parameters, $\lambda_{T_{1}T_{r}}$ and $\zeta_{T_{\beta }}$, and used them in a mathematical model to predict the response rate to treatment with anti-PD-1 as single agent and in combination with anti-TGF-*β*.

Our simulations, in Figs 2 and 4, show agreement with the experimental results (in mice) reported in [15, 22, 23]. We also show, in Fig 3, that under treatment with anti-PD-1 alone, as the parameters $\lambda_{T_{1}T_{r}}$ and $\zeta_{T_{\beta }}$ increase the progression of cancer increases, while treatment does not result in progression of cancer if either $\lambda_{T_{1}T_{r}}$ or $\zeta_{T_{\beta }}$ is small.

The parameters $\lambda_{T_{1}T_{r}}$ and $\zeta_{T_{\beta }}$ can be viewed as biomarkers, predicting the following:

(i) for any combination $\left(\gamma_{A_{\beta }},\gamma_{A_{1}}\right)$, the efficacy increases when $\lambda_{T_{1}T_{r}}$ and $\zeta_{T_{\beta }}$ are decreased.
(ii) For large values of $\lambda_{T_{1}T_{r}}$ and $\zeta_{T_{\beta }}$, tumor progression is likely to occur.
(iii) For small values of $\lambda_{T_{1}T_{r}}$ and $\zeta_{T_{\beta }}$, the efficacy increases as $\gamma_{A_{1}}$ and $\gamma_{A_{\beta }}$ are increased.

We also found that while efficacy always inceases when $\gamma_{A_{\beta }}$ is increased, there are regions in the $\left(\gamma_{A_{\beta }},\gamma_{A_{1}}\right)$-plane such that efficacy is decreased as $\gamma_{A_{1}}$ increases: these regions consist of points $\left\{(\gamma_{A_{\beta }},\gamma_{A_{1}}):\;\gamma_{A_{1}}^{-}<\gamma_{A_{1}}<\gamma_{A_{1}}^{+}\right\}$, where $\gamma_{A_{1}}^{-}$ and $\gamma_{A_{1}}^{+}$ depend on $\gamma_{A_{\beta }}$.

The mathematical model presented in this paper has several limitations:

1. We assumed that the densities of immature, or naive, immune cells remain constant throughout the progression of the cancer and that dead cells are quickly removed from the tumor.
2. In estimating production parameters we made a steady state assumption in some of the differential equations.
3. Although our mathematical model does not presume any geometric form of the tumor, for simplicity, the simulations have been carried out only in the case of spherical tumor. We note however that spherical cancer models have been used in research as an intermediate between *in vitro* cancer line cultures and *in vivo* cancer [54]. Furthermore, spheroids mirror the 3D cellular context and therapeutically relevant pathophysiological gradient of *in vivo* tumors [55].

Biomarkers are characteristics of the body and they are critical in order to diagnose a disease and/or to measure the effect of a drug on the patient. In the present paper, based on mice experiments, we developed a mathematical model which demonstrates, depending on two parameters, how primary resistance to anti-PD-1 can be overcome by anti-TGF-beta. These parameters may serve as new cancer biomarkers, but our results will first need to be validated by clinical studies.

## Supporting information

S1 File TGF-*β* inhibition can overcome cancer primary resistance to PD-1 blockade: A mathematical model. Model equations (Section 1 in S1 File), parameter estimates (Section 2 in S1 File), parameter sensitivity analysis (Section 3 in S1 File), numerical methods used (Section 4 in S1 File) and the parameter values (Tables 1 and 2 in S1 File).
(PDF)
