# Supplementary material for: TGF-β inhibition can overcome cancer primary resistance to PD-1 blockade: A mathematical model
Source: PLoS One. 2021 Jun 1;16(6):e0252620. doi: 10.1371/journal.pone.0252620 (PMC8168900; doi:10.1371/journal.pone.0252620)
Supplement: S1 File — Model equations (Section 1 in S1 File), parameter estimates (Section 2 in S1 File), parameter sensitivity analysis (Section 3 in S1 File), numerical methods used (Section 4 in S1 File) and the parameter values (Tables 1 and 2 in S1 File). (PDF) [file pone.0252620.s001.pdf]

TGF-β inhibition can overcome cancer primary resistance to PD-1 blockade: a mathematical model

Nourridine Siewe[1*], Avner Friedman[2]

1 School of Mathematical Sciences, College of Science, Rochester Institute of Technology, Rochester, New York, U.S.A.
2 Mathematical Biosciences Institute & Department of Mathematics, The Ohio State University, Columbus, Ohio, U.S.A.

* nxssma@rit.edu

1 Model Equations

We assume that the combined densities of cells within the tumor remains constant in space and time:

$$C + D + M_1 + M_2 + T_1 + T_8 + T_r = \theta, \tag{1}$$

for some constant $\theta > 0$. We assume that the densities of immature dendritic cells and naive CD4$^+$ and CD8$^+$ T cells remain constant throughout the tumor tissue. Under Assumption (1), proliferation of cancer cells and immigration of immune cells into the tumor, give rise to internal pressure which results in cells movement. We assume that all the cells move with the same velocity, $\mathbf{u}$; $\mathbf{u}$ depends on space and time and will be taken in units of cm/day. We assume that cytokines and anti-tumor drugs are diffusing within the tumor, and that also cells undergo diffusion (i.e., dispersion).

Equation for tumor cells (C) We write the equation for C in the following form:

$$\frac{\partial C}{\partial t} + \nabla \cdot (\mathbf{u}C) - \delta_C \nabla^2 C = \underbrace{\lambda_C C \left(1 - \frac{C}{C_M}\right)}_{\text{Growth of cancer cells}} - \underbrace{\frac{\mu_{T_8 C}}{1 + \zeta_{T_\beta} T_\beta} T_8 C}_{\text{killing by } T_8} - \underbrace{\mu_C C}_{\text{death}}. \tag{2}$$

Equation for dendritic cells (D) Dendritic cells are activated by the high mobility group box 1 (HMGB-1) expressed on necrotic cancer cells [1, 2]. We assume that the density of HMGB-1 is proportional to the density of C. Hence, the activation rate of immature dendritic cells, with density D_0, is proportional $\frac{C}{K_C + C}$, for some constant K_C. The dynamics of dendritic cells is given by

$$\frac{\partial D}{\partial t} + \nabla \cdot (\mathbf{u}D) - \delta_D \nabla^2 D = \underbrace{\lambda_{DC} D_0 \frac{C}{K_C + C}}_{\text{activation by HMGB-1}} - \underbrace{\mu_D D}_{\text{death}}, \tag{3}$$

where δ_D is the diffusion coefficient and μ_D is the death rate of dendritic cells, and the activation rate λ_{DC} is a constant.

Equation for M1 macrophages (M_1) The equation for M1 macrophages has the following form:

$$\frac{\partial M_1}{\partial t} + \nabla\cdot(\mathbf{u}M_1) - \delta_M\nabla^2 M_1 = \underbrace{\lambda_{M_1}M_0\frac{P}{K_P+P}}_{\text{activation by CCL2}} - \underbrace{\nabla\cdot(\chi_P M_1\nabla P)}_{\text{chemoattraction by CCL2}} + \underbrace{\lambda_{M_2M_1}M_2\frac{I_{12}}{K_{I_{12}}+I_{12}}}_{M_2\to M_1 \text{ by IL-12}} - \underbrace{\lambda_{M_1M_2}M_1\frac{T_\beta}{K_{T_\beta}+T_\beta}}_{M_1\to M_2 \text{ by TGF-}\beta} - \underbrace{\mu_{M_1}M_1}_{\text{death}}. \tag{4}$$

Equation for MDSCs (M_2). The equation for M_2 is given by:

$$\frac{\partial M_2}{\partial t} + \nabla\cdot(\mathbf{u}M_2) - \delta_M\nabla^2 M_2 = \underbrace{\lambda_{M_2}M_0\frac{P}{K_P+P}}_{\text{activation by CCL2}} - \underbrace{\nabla\cdot(\chi_P M_2\nabla P)}_{\text{chemoattratced by CCL2}} - \underbrace{\lambda_{M_2M_1}M_2\frac{I_{12}}{K_{I_{12}}+I_{12}}}_{M_2\to M_1 \text{ by IL-12}} + \underbrace{\lambda_{M_1M_2}M_1\frac{T_\beta}{K_{T_\beta}+T_\beta}}_{M_1\to M_2 \text{ by TGF-}\beta} - \underbrace{\mu_{M_2}M_2}_{\text{death}}. \tag{5}$$

Equation for CD4$^+$ T/Th1 cells (T_1). T_1 satisfies the following equation:

$$\frac{\partial T_1}{\partial t} + \underbrace{\nabla\cdot(\mathbf{u}T_1)}_{\text{advection}} - \underbrace{\delta_T\nabla^2 T_1}_{\text{diffusion}} = \tag{6}$$
$$\Bigg(\lambda_{T_1I_{12}}T_{10}\underbrace{\left(1+\frac{T_\beta}{K_{T_\beta}+T_\beta}\right)}_{T_\beta\text{-augmented activation}}\underbrace{\frac{I_{12}}{K_{I_{12}}+I_{12}}}_{\text{activation by IL-12}}\cdot\underbrace{\frac{1}{1+I_{10}/\hat{K}_{TI_{10}}}}_{\text{inhibition by IL-10}}\cdot\underbrace{\frac{1}{1+T_r/\hat{K}_{TT_r}}}_{\text{inhibition by Tregs}} + \underbrace{\lambda_{T_1I_2}T_1\frac{I_2}{K_{I_2}+I_2}}_{\text{IL-2-induced proliferation}}\Bigg)\times\underbrace{\frac{1}{1+Q/\hat{K}_{TQ}}}_{\text{inhibition by } Q} - \underbrace{\lambda_{T_1T_r}T_1\frac{Q}{K_Q+Q}}_{Q\text{-induced } T_1\to T_r \text{ transition}} - \underbrace{\mu_{T_1}T_1}_{\text{death}}.$$

Equation for activated CD8$^+$ T cells (T_8). T_8 satisfies the following equation:

$$\frac{\partial T_8}{\partial t} + \nabla\cdot(\mathbf{u}T_8) - \delta_T\nabla^2 T_8 = \tag{7}$$
$$\Bigg(\lambda_{T_8I_{12}}T_{80}\underbrace{\left(1+\frac{T_\beta}{K_{T_\beta}+T_\beta}\right)}_{T_\beta\text{-augmented activation}}\underbrace{\frac{I_{12}}{K_{I_{12}}+I_{12}}}_{\text{activation by IL-12}}\cdot\underbrace{\frac{1}{1+I_{10}/\hat{K}_{TI_{10}}}}_{\text{inhibition by IL-10}}\cdot\underbrace{\frac{1}{1+T_r/\hat{K}_{TT_r}}}_{\text{inhibition by Tregs}} + \underbrace{\lambda_{T_8I_2}T_8\frac{I_2}{K_{I_2}+I_2}}_{\text{IL-2-induced proliferation}}\Bigg)\times\underbrace{\frac{1}{1+Q/\hat{K}_{TQ}}}_{\text{inhibition by } Q} - \underbrace{\mu_{T_8}T_8}_{\text{death}}.$$

Equation for Tregs (T_r). We write the following equation for T_r:

$$
\begin{aligned}
&\frac{\partial T_r}{\partial t} + \nabla\cdot(\mathbf{u}T_r) - \delta_T\nabla^2 T_r = \qquad (8)\\
&\underbrace{\lambda_{T_rT_\beta}T_{10}\frac{T_\beta}{K_{T_\beta}+T_\beta}}_{T_\beta\text{-enhanced naive T cells activation}} + \underbrace{\lambda_{T_1T_r}T_1\frac{Q}{K_Q+Q}}_{Q\text{-induced } T_1\to T_r \text{ transition}} + \underbrace{\lambda_{T_rI_2}T_r\frac{I_2}{K_{I_2}+I_2}}_{\text{IL-2-induced proliferation}}\\
&- \underbrace{\nabla\cdot(\chi_P T_r\nabla P)}_{\text{chemoattraction by CCL2/MCP-1}} - \underbrace{\mu_{T_r}T_r}_{\text{death}}.
\end{aligned}
$$

Equation for IL-2 (I_2). Cytokine IL-2 is produced by activated CD4$^+$ T cells [3]. Hence,

$$\frac{\partial I_2}{\partial t} - \delta_{I_2}\nabla^2 I_2 = \underbrace{\lambda_{I_2T_1}T_1}_{\text{secretion by CD4}^+\text{ T cells}} - \underbrace{\mu_{I_2}I_2}_{\text{degradation}}. \qquad (9)$$

Equation for IL-10 (I_{10}). Cytokine IL-10 is produced by cancer cells and MDSCs [4]. Hence IL-10 satisfies the following equation:

$$\frac{\partial I_{10}}{\partial t} - \delta_{I_{10}}\nabla^2 I_{10} = \underbrace{\lambda_{I_{10}C}C + \lambda_{I_{10}M_2}M_2}_{\text{secretion by } C \text{ and } M_2} - \underbrace{\mu_{I_{10}}I_{10}}_{\text{degradation}}. \qquad (10)$$

Equation for IL-12 (I_{12}). The pro-inflammatory cytokine IL-12 is secreted by activated dendritic cells [3, 5], so that

$$\frac{\partial I_{12}}{\partial t} - \delta_{I_{12}}\nabla^2 I_{12} = \underbrace{\lambda_{I_{12}D}D}_{\text{secretion by DCs}} - \underbrace{\mu_{I_{12}}I_{12}}_{\text{degradation}}. \qquad (11)$$

Equation for CCL2/MCP-1 (P). Cytokine CCL2 is produced by cancer cells [6–8], so that

$$\frac{\partial P}{\partial t} - \delta_P\nabla^2 P = \underbrace{\lambda_{PC}C}_{\text{secretion by cancer cells}} - \underbrace{\mu_P P}_{\text{degradation}}. \qquad (12)$$

Equation for TGF-β (T_β). The cytokine TGF-β is produced by tumor cells [4], MDSCs [9–11] and Tregs [12]. Hence,

$$\frac{\partial T_\beta}{\partial t} - \delta_{T_\beta}\nabla^2 T_\beta = \underbrace{\lambda_{T_\beta C}C + \lambda_{T_\beta M_2}M_2 + \lambda_{T_\beta T_r}T_r}_{\text{secretion by } C,\ M_2 \text{ and } T_r} - \underbrace{\mu_{T_\beta}T_\beta}_{\text{degradation}}.$$

When anti-TGF-β drug is applied, TGF-β is depleted at a rate proportional to A_β, and the equation for T_β takes the following form:

$$\frac{\partial T_\beta}{\partial t} - \delta_{T_\beta}\nabla^2 T_\beta = \underbrace{\lambda_{T_\beta C}C + \lambda_{T_\beta M_2}M_2 + \lambda_{T_\beta T_r}T_r}_{\text{secretion by } C,\ M_2 \text{ and } T_r} - \underbrace{\mu_{T_\beta}T_\beta}_{\text{degradation}} - \underbrace{\mu_{A_\beta T_\beta}T_\beta A_\beta}_{\text{depletion by anti-TGF-}\beta}, \qquad (13)$$

where $\mu_{A_\beta}T_\beta$ is the depletion rate of T_β.

Equations for PD-1 (P_D), PD-L1 (P_L) and PD-1/PD-L1 (Q). PD-1 is expressed on the membrane of activated CD4$^+$ T cells, activated CD8$^+$ T cells and Tregs. We assume that the number of PD-1 per cell is the same for CD4$^+$ and CD8$^+$ T cells, but smaller, by a factor ε_T, for Treg cells. If we denote by ρ_{P_D} the ratio between the mass of the PD-1 proteins in one T cell to the mass of this cell, then

$$P_D = \rho_{P_D}(T_1 + T_8 + \varepsilon_T T_r).$$

The coefficient ρ_{P_D} is constant when no anti-PD-1 drug is administered. In this case, to a change in $T = T_1 + T_8 + \varepsilon_T T_r$, given by $\partial T/\partial t$, there corresponds a change of P_D, given by $\rho_{P_D}\partial T/\partial t$. For the same reason, $\nabla \cdot (\mathbf{u}P_D) = \rho_{P_D}\nabla \cdot (\mathbf{u}T)$ and $\nabla^2 P_D = \rho_{P_D}\nabla^2 T$ when no anti-PD-1 drug is injected. Hence, P_D satisfies the equation:

$$\frac{\partial P_D}{\partial t}+\nabla\cdot(\mathbf{u}P_D)-\delta_T\nabla^2 P_D = \frac{\partial(T_1+T_8+\varepsilon_T T_r)}{\partial t}+\nabla\cdot(\mathbf{u}(T_1+T_8+\varepsilon_T T_r))-\delta_T\nabla^2(T_1+T_8+\varepsilon_T T_r).$$

Recalling Eqs. (6)–(8) for $T_1,\ T_8$ and T_r, we get

$$\begin{aligned}
&\frac{\partial P_D}{\partial t} + \nabla\cdot(\mathbf{u}P_D) - \delta_T\nabla^2 P_D = \qquad (14)\\
&\quad \rho_{P_D}\Bigg\{ \Bigg[(\lambda_{T_1 I_{12}}T_{10} + \lambda_{T_8 I_{12}}T_{80})\left(1+\frac{T_\beta}{K_{T_\beta}+T_\beta}\right)\frac{I_{12}}{K_{I_{12}}+I_{12}}\cdot\frac{1}{1+I_{10}/\hat{K}_{TI_{10}}}\times\\
&\quad \frac{1}{1+T_r/\hat{K}_{TT_r}} + (\lambda_{T_1 I_2}T_1 + \lambda_{T_8 I_2}T_8)\frac{I_2}{K_{I_2}+I_2}\Bigg]\frac{1}{1+Q/\hat{K}_{TQ}}\\
&\quad - \lambda_{T_1 T_r}T_1\frac{Q}{K_Q+Q} + \varepsilon_T\left(\lambda_{T_r T_\beta}T_{10}\frac{T_\beta}{K_{T_\beta}+T_\beta} + \lambda_{T_1 T_r}T_1\frac{Q}{K_Q+Q} + \lambda_{T_r I_2}T_r\frac{I_2}{K_{I_2}+I_2}\right)\\
&\quad - [(\mu_{T_1}T_1 + \mu_{T_8}T_8 + \varepsilon_T\mu_{T_r}T_r) + \varepsilon_T\chi_P\nabla\cdot(T_r\nabla P)]\Bigg\}.
\end{aligned}$$

When anti-PD-1 drug is applied, PD-1 is depleted at a rate proportional to A_1, and, in this case, the ratio $P_D/(T_1 + T_8 + \varepsilon_T T_r)$ may change. In order to include in the model both cases of with and without anti-PD-1, we replace ρ_{P_D} in Eq. (14) by $P_D/(T_1 + T_8 + \varepsilon_T T_r)$. Hence,

$$\begin{aligned}
&\frac{\partial P_D}{\partial t} + \nabla\cdot(\mathbf{u}P_D) - \delta_T\nabla^2 P_D =\\
&\frac{P_D}{T_1+T_8+\varepsilon_T T_r}\Bigg\{ \Bigg[(\lambda_{T_1 I_{12}}T_{10} + \lambda_{T_8 I_{12}}T_{80})\left(1+\frac{T_\beta}{K_{T_\beta}+T_\beta}\right)\frac{I_{12}}{K_{I_{12}}+I_{12}}\cdot\frac{1}{1+I_{10}/\hat{K}_{TI_{10}}}\times\\
&\frac{1}{1+T_r/\hat{K}_{TT_r}} + (\lambda_{T_1 I_2}T_1 + \lambda_{T_8 I_2}T_8)\frac{I_2}{K_{I_2}+I_2}\Bigg]\frac{1}{1+Q/\hat{K}_{TQ}}\\
&- \lambda_{T_1 T_r}T_1\frac{Q}{K_Q+Q} + \varepsilon_T\left(\lambda_{T_r T_\beta}T_{10}\frac{T_\beta}{K_{T_\beta}+T_\beta} + \lambda_{T_1 T_r}T_1\frac{Q}{K_Q+Q} + \lambda_{T_r I_2}T_r\frac{I_2}{K_{I_2}+I_2}\right)\\
&- [(\mu_{T_1}T_1 + \mu_{T_8}T_8 + \varepsilon_T\mu_{T_r}T_r) + \varepsilon_T\chi_P\nabla\cdot(T_r\nabla P)]\Bigg\}\\
&- \underbrace{\mu_{P_D A_1}P_D A_1}_{\text{depletion by anti-PD-1}},
\end{aligned}$$

where μ_{PDA_1} is the depletion rate of PD-1 by anti-PD-1.

We assume that the number of PD-L1 proteins in one T_1 cell is the same as for one T_8 cell, and denote by ρ_{P_L} the ratio of the mass of all the PD-L1 proteins in one T_1 cell

to the mass of one cell. We assume that this ratio is smaller for the PD-L1 proteins on MDSC and cancer cells, by factors ε_M and ε_C, respectively, so that

$$P_L = \rho_{P_L}(T_1 + T_8 + \varepsilon_M M_2 + \varepsilon_C C). \tag{15}$$

PD-L1 from T cells or cancer cells combines with PD-1 on the plasma membrane of T cells, forming a complex PD-1/PD-L1 (Q) on the T cells [13, 14]. Denoting the association and disassociation rates of Q by $\alpha_{P_D P_L}$ and μ_Q, respectively, we write

$$P_D + P_L \underset{\mu_Q}{\overset{\alpha_{P_D P_L}}{\rightleftharpoons}} Q.$$

Since the half-life of Q is less than 1 second (i.e., 1.16×10^{-5} day) [15], we may approximate the dynamical equation for Q by the steady state equation $\alpha_{P_D P_L} = \mu_Q Q$, or

$$Q = \sigma P_D P_L, \tag{16}$$

where $\sigma = \alpha_{P_D P_L}/\mu_Q$.

Equation for cells velocity (u). We assume that all cells have approximately the same diffusion coefficient. Adding Eqs. (2)–(8) and using Eq, (1), we get

$$\theta \times \nabla \cdot \mathbf{u} = \sum_{j=2}^{8} [\text{right-hand side of Eq. } (2.j)]. \tag{17}$$

To simplify the computations, we assume that the tumor is spherical and that all the densities and concentrations are radially symmetric, that is, functions of (r, t), $0 \le r \le R(t)$, where $r = R(t)$ is the boundary of the tumor, and that $\mathbf{u} = u(r,t)\mathbf{e}_r$, where $\mathbf{e}_r$ is the unit radial vector.

Boundary conditions. We assume that the inactive CD4$^+$ and CD8$^+$ T cells that migrated from the lymph nodes into the tumor microenvironment have constant densities $\hat{T}_1$ and $\hat{T}_8$, respectively, at the tumor boundary, and that they are activated by IL-12 upon entering the tumor. We then have the following conditions at the tumor boundary:

$$\begin{aligned} \frac{\partial T_1}{\partial t} &+ \sigma_0 \frac{I_{12}}{K_{I_{12}} + I_{12}}(T_1 - \hat{T}_1) = 0, \\ \frac{\partial T_8}{\partial r} &+ \sigma_0 \frac{I_{12}}{K_{I_{12}} + I_{12}}(T_8 - \hat{T}_8) = 0 \quad \text{at } r = R(t). \end{aligned} \tag{18}$$

We impose no-flux boundary condition on all the remaining variables:

$$\text{No flux for } M_1,\ M_2,\ D,\ T_r,\ C,\ P,\ I_{10},\ I_{12},\ T_\beta,\ P_D,\ A_1 \text{ and } A_\beta \text{ at } r = R(t).$$

It is tacitly assumed here that the receptors PD-1 and ligands PD-L1 become active only after the T cells are already inside the tumor.

Initial conditions (at $t = t_0$). We take the initial values of cells (in units of g/cm^3) as follows:

$$\begin{aligned} M_1 &= 2.5 \times 10^{-3},\ M_2 = 3.2 \times 10^{-3},\ D = 6.3 \times 10^{-5}, \\ T_1 &= 1.5 \times 10^{-5},\ T_8 = 2.14 \times 10^{-5},\ T_r = 1.1 \times 10^{-3},\ C = 8.2 \times 10^{-2}. \end{aligned} \tag{19}$$

Then, by Eq. (1), $\theta = 0.632$ in Eq. (17).

We assume that initially $A_1 = 0$ and $A_\beta = 0$, and

$$\begin{aligned} P = 1.3 \times 10^{-7},\ I_{10} = 2.5 \times 10^{-9},\ I_{12} = 1.4 \times 10^{-10}, \\ T_\beta = 1.4 \times 10^{-8},\ P_D = 5 \times 10^{-11},\ P_L = 6 \times 10^{-8}. \end{aligned} \tag{20}$$

Other nearby choices of initial conditions do not affect the simulations of the model after a few days.

2 Parameter Estimates

Half-saturation. In an expression of the form $Y \dfrac{X}{K_X + X}$ where Y is activated by X, the parameter K_X is called the half-saturation of X. We denote by X^0 the average concentration of X, and assume that

$$\frac{X^0}{K_X + X^0}$$

to be not too close to 0 or to 1, and, for simplicity, take it to be 1/2, so that

$$K_X = X^0. \tag{21}$$

Estimate for K_D. Vescovi et al. [16] measured the density of plasmacytoid dendritic cells (PDCs) in various cohorts of patients with advanced cutaneous melanomas. They collected blood samples from 29 metastatic melanoma patients and plotted the distributions of PDCs from these patients. The authors reported that the density of PDCs ranges between almost zero to 400 cells/mm^3, with an average of approximately 80 cells/mm^3, or equivalently 80×10^3 cells/cm^3. In the sequel we assume that the mass of 1 cell is approximately 5×10^{-10} g [17]. Hence,

$$K_D = D^0 = 80 \times 10^3 \times 5 \times 10^{-10} = 4 \times 10^{-5} \text{ g/cm}^3.$$

Estimate for K_{T_8}. Lisiero et al. [18] measured the level of CD8$^+$ T cells in a melanoma model in mice, under IL-12 priming. They reported levels of $7 - 10 \times 10^5$ cells/cm^3 after three days. Since the mass of one cell is approximately 5×10^{-10} g, we take

$$K_{T_8} = T_8^0 = 10^6 \times 5 \times 10^{-10} = 5 \times 10^{-4} \text{ g/cm}^3.$$

Estimates for $K_{I_{10}}$ and $\hat{K}_{TI_{10}}$. The expression of IL-10 in human malignant melanoma was investigated by Kruger-Krasagakes et al. [19]. They found that the level of IL-10 ranged between $0.6 - 6 \times 10^{-9}$ g/cm^3. We take

$$K_{I_{10}} = 5 \times 10^{-9} \text{ g/cm}^3 \quad \text{and} \quad \hat{K}_{I_{10}} = 5 \times 10^{-9} \text{ g/cm}^3.$$

Estimate for $K_{I_{12}}$. Jafarzadeh et al. [20] evaluated the circulating levels of IL-12 in patients with breast cancer. Their study included healthy humans and patients in different stages of breast cancer (stages 1–4) and found that the circulating level of IL-12 in stage 4 was approximately $\sim$100 pg/cm^3. We assume that advanced level of melanoma corresponds to stage 4 breast cancer, and take

$$K_{I_{12}} = I_{12}^0 = 10^{-10} \text{g/cm}^3.$$

Estimate for K_{T_β}. The average level of TGF-β in primary melanoma tumor line is 36 pg/10^5 cells [21], or equivalently 36×10^{-12} g/($10^5 \times 5 \times 10^{-10}$ cm^3), assuming that the volume of 1 cell is 5×10^{-10} g [17]. Hence

$$K_{T_\beta} = T_\beta^0 = 7.2 \times 10^{-9} \text{ g/cm}^3.$$

Estimate of $\hat{K}_{TQ}$ and K_Q. Denoting the association and disassociation rates of the complex Q=PD-1/PD-L1 by $\alpha_{P_DP_L}$ and d_Q, respectively, we can write

$$P_D + P_L \underset{\mu_Q}{\overset{\alpha_{P_DP_L}}{\rightleftharpoons}} Q.$$

The half-life of Q is less than 1 second (i.e., 1.16×10^{-5} day) [15], so that μ_Q is very large. Hence, we may approximate the dynamical equation for Q by the steady-state equation

$$\alpha_{P_DP_L} P_D P_L = \mu_Q Q, \qquad \text{or} \qquad Q = \sigma P_D P_L,$$

where $\sigma = \alpha_{P_DP_L}/\mu_Q$. We can then write the inhibition of Th1 and CD8$^+$ T cells by Q in the form

$$\frac{1}{1 + Q/K_{TQ}} = \frac{1}{1 + P_D P_L/\hat{K}_{TQ}},$$

and take, as in [22],

$$\hat{K}_{TQ} = 4.86 \times 10^{-20} \text{ g}^2/\text{cm}^6.$$

We assume that

$$K_Q = 4.86 \times 10^{-20} \text{ g}^2/\text{cm}^6.$$

Estimates for M_0, D_0 and T_0. We estimate the levels of macrophages, dendritic cells and T cells in the mouse liver, and assume that these levels are similar in other mouse organs.

We note that in mouse liver there are 1.5×10^7 macrophages [23], 10^7 T cells [24] and 7×10^6 DCs [25]. The weight of a mouse liver is 2 grams [26,27], which corresponds, approximately, to a volume of 2 cm^3. Hence the density of macrophages in mouse liver is 7.5×10^6 cells/cm^3, the density of T cells is 5×10^6 cells/cm^3, and the density of DC is 3.5×10^6 cells/cm^3.

Since the mass of 1 cell is assumed to be 5×10^{-10} g, we get

$$M_0 = 7.5 \times 10^6 \times 5 \times 10^{-10} = 1.5 \times 10^{-3} \text{ g/cm}^3. \tag{22}$$

Similarly,

$$T_{10} = 5 \times 10^6 \times 5 \times 10^{-10} = 2.5 \times 10^{-3} \text{ g/cm}^3. \tag{23}$$

We take

$$T_{80} = T_{10}/2 = 1.25 \times 10^{-3} \text{ g/cm}^3. \tag{24}$$

We assume that the mass density of DC is smaller than that of M_0, and take

$$D_0 = \frac{1}{10} M_0 = 1.5 \times 10^{-4} \text{g/cm}^3. \tag{25}$$

Estimates for $\hat{T}_1$ and $\hat{T}_8$. We take

$$\hat{T}_1 = T_{10} = 2.5 \times 10^{-3} \text{ g/cm}^3, \quad \hat{T}_8 = T_{80} = 1.25 \times 10^{-3} \text{ g/cm}^3.$$

Estimates for the diffusion coefficients of cytokines and drugs. Young [28] established the following formula for estimating the diffusion coefficient δ_p of a protein p:

$$\delta_p = \frac{\beta}{M_p^{1/3}}, \tag{26}$$

where M_p is the molecular weight of p and β is a constant. Since for V=VEGF $M_V = 24$ kDa [29] and $\delta_V = 8.64 \times 10^{-2}$ cm^2 d^{-1} [30],

$$\beta = 8.64 \times 10^{-2} \times (24)^{1/3} = 0.25 \text{ cm}^2 \text{ d}^{-1} \text{ (kDa)}^{1/3}.$$

From $M_P = 11$ kDa [29], $M_{I_2} = 16$ kDa [29], $M_{I_{10}} = 20.5$ kDa [29], $M_{I_{12}} = 37$ kDa [29] and $M_{T_\beta} = 4.76$ kDa [31]. We deduce that $\delta_P = 0.11$ cm^2 d^{-1}, $\delta_{I_2} = 9.92 \times 10^{-2}$ cm^2 d^{-1} $\delta_{I_{10}} = 9.13 \times 10^{-2}$ cm^2 d^{-1}, $\delta_{I_{12}} = 7.5 \times 10^{-2}$ cm^2 d^{-1}, $\delta_{T_\beta} = 14.8 \times 10^{-2}$ cm^2 d^{-1}.

We assume that the formula (26) can be applied also to drugs; since $M_{A_1} = 32$ kDa [29,32] and $M_{A_\beta} = 44$ kDa [33], we get: $\delta_{A_1} = 7.87 \times 10^{-2}$ cm^2 d^{-1} and $\delta_{A_\beta} = 4.7 \times 10^{-2}$ cm^2 d^{-1}.

Estimates for the diffusion coefficient of cells, $\delta_X, \ X \in \{C, D, M_1, M_2, T_1, T_8, T_r\}$. We take the diffusion coefficient of macrophages to be $\delta_M = 8.64 \times 10^{-7}$ cm^2 d^{-1} [34], and assume that all other cell types have, approximately, the same diffusion coefficient, so that

$$\delta_X = 8.64 \times 10^{-7} \text{ cm}^2 \text{ d}^{-1}, \text{ for } X = C,\ D,\ M_1,\ M_2,\ T_1,\ T_8,\ T_r.$$

Estimate for λ_C. The growth rate of human melanoma is $\lambda_C^h = 0.616$ d^{-1} [22]. We assume that melanoma grows faster in mice than in humans, and take $\lambda_C = 1.2\lambda_C^h = 0.739$ d^{-1}.

Estimate for $\mu_{P_D A_1}$. The parameter $\mu_{P_D A_1}$ was estimated, in [22], to be 6.04×10^6 cm^3/g·d, within a factor of 2. We take

$$\mu_{P_D A_1} = 1.2 \times 10^7 \text{ cm}^3/\text{g}\cdot\text{d}.$$

Estimates for μ_{M_1} and μ_{M_2}. The half-life of M_1 macrophages is 3 weeks and of M_2 macrophages is 1 week [35]. Hence,

$$\mu_{M_1} = \frac{\ln 2}{21 \text{ d}} = 3.3 \times 10^{-2} \text{ d}^{-1}, \text{ and } \mu_{M_2} = \frac{\ln 2}{7 \text{ d}} = 9.9 \times 10^{-2} \text{ d}^{-1}.$$

Estimate for μ_{T_r}. The half-life of the regulatory T cells is approximately 2.7 days [36]. Hence,

$$\mu_{T_r} = \frac{\ln 2}{2.7 \text{ d}} = 0.25 \text{ d}^{-1}.$$

Estimate for μ_{T_β}. The half-life of active TGF-β is 2–3 minutes [37,38]; taking it approximately 2.5 minutes, we get

$$\mu_{T_\beta} = \frac{\ln 2}{1.74 \times 10^{-3} \text{ d}} = 399.25 \text{ d}^{-1}.$$

Estimate for μ_P. The half-life of CCL2 ranges between 3-9.5h [39]. We take

$$\mu_P = \frac{\ln 2}{0.167 \text{ d}} = 4.16 \text{ d}^{-1}.$$

Estimate for μ_Q. The half-life of Q is less than 1 second (i.e., 1.16×10^{-5} day) [15]. We take

$$\mu_Q = \frac{\ln 2}{1.16 \times 10^{-5} \text{ d}} = 6 \times 10^4 \text{ d}^{-1}.$$

Estimate for μ_{A_β}. The half-life of anti-TGF-β ranges between 8.6h (galunisertib plasma) and 10h ([^{14}C]-galunisertib) [40]. We take it to be 9.3h (0.39 days), so that

$$\mu_{A_\beta} = \frac{\ln 2}{0.39 \text{ d}} = 1.78 \text{ d}^{-1}.$$

Estimate for $\lambda_{I_{10}M_2}$. The secretion rate of IL-10 by macrophages is 5.3×10^{-5} per day [41]. Hence,

$$\lambda_{I_{10}M_2} = 5.3 \times 10^{-4} \text{ per day.}$$

Estimates for ε_C, ε_M and ε_T. The expression of PD-L1 in cancer cells is cancer-specific. In line with [22, 42], we take

$$\varepsilon_C = 0.01.$$

We take the PD-L1 expression in MDSC such that

$$\varepsilon_M = 0.01,$$

and the PD-1 expression in Treg such that

$$\varepsilon_T = 0.01.$$

Estimates by equation.

Eq. (2)**:** We use the steady state equation

$$\lambda_C C(1 - C/C_M) - (\mu_{T_8C}/(1 + \zeta_{T_\beta} T_\beta))T_8 C = 0,$$

with $\lambda_C = 0.739$ d^{-1}, $\zeta_{T_\beta} = 4 \times 10^7$ cm^3/g, $\mu_C = 0.17$ [22], $K_C = 0.4$ g/cm^3, $C_M = 0.8$ g/cm^3 [43, 44], $K_{T_\beta} = 7.2 \times 10^{-9}$ g/cm^3, and $K_{T_8} = 5 \times 10^{-4}$ g/cm^3. Hence,

$$\mu_{T_8C} = 1347.02 \text{ cm}^3/\text{g·d}.$$

Eq. (3)**:** We use the steady state equation

$$\lambda_{DC} D_0/2 - \mu_D D = 0,$$

where $\mu_D = 0.1$ d^{-1} [45], $D_0 = 1.5 \times 10^{-4}$ g/cm^3, and $K_D = 4 \times 10^{-5}$ g/cm^3. Hence,

$$\lambda_{DC} = 0.053 \text{ d}^{-1}.$$

Eq. (6)**:** The efficacy of anti-PD-1 decreases as $\lambda_{T_1T_r}$ increases. We take $\lambda_{T_1T_r} = 10^3$ d^{-1} in HPD, and $\lambda_{T_1T_r} = 6.24$ d^{-1} otherwise. The choice of 6.24 d^{-1} was made by using the steady state equation

$$\frac{\lambda_{T_1I_{12}} T_{10}(1 + /2)/8 + \lambda_{T_1I_2} K_{T_1}}{2} - \lambda_{T_1T_r} K_{T_1}/2 - \mu_{T_1} K_{T_1} = 0,$$

with the estimates $\lambda_{T_1I_{12}} = 27.26$ d^{-1} [43], $\lambda_{T_1I_2} = 0.25$ d^{-1} [43], $\mu_{T_1} = 0.197$ d^{-1} [46–48], $T_{10} = 2.5 \times 10^{-3}$ g/cm^3, and $K_{T_1} = 2 \times 10^{-3}$ g/cm^3 [43].

Eq. (7)**:** We use the steady state equation

$$(\lambda_{T_8I_{12}} T_{80}(1 + 1/2)/8 + \lambda_{T_8I_2} T_8/2)/2 - \mu_{T_8} T_8 = 0,$$

where $\lambda_{T_8I_2} = 0.25$ d^{-1} [43], $\mu_{T_8} = 0.18$ [46–48], $T_{80} = 1.25 \times 10^{-3}$ g/cm^3, and $K_{T_8} = 5 \times 10^{-4}$ g/cm^3. Hence,

$$\lambda_{T_8I_{12}} = 0.486 \text{ d}^{-1}.$$

Eq. (10)**:** We assume that MDSCs produce more IL-10 than cancer cells, and take

$$\lambda_{I_{10}M_2}M_2 = 5\lambda_{I_{10}C}C. \quad (27)$$

Then, from the steady state

$$\lambda_{I_{10}C}C + \lambda_{I_{10}M_2}M_2 - \mu_{I_{10}}I_{10} = 0,$$

where $\mu_{I_{10}} = 8.32\ \text{d}^{-1}$ [22], $K_{I_{10}} = 5 \times 10^{-9}\ \text{g/cm}^3$, $K_C = 0.4\ \text{g/cm}^3$, and $K_{M_2} = 3.2 \times 10^{-3}\ \text{g/cm}^3$ [43], we get

$$\lambda_{I_{10}C} = 1.73 \times 10^{-8}\ \text{d}^{-1} \quad \text{and} \quad \lambda_{I_{10}M_2} = 1.1 \times 10^{-5}\ \text{d}^{-1}.$$

Eq. (11)**:** From the steady state equation

$$\lambda_{I_{12}D}D - \mu_{I_{12}}I_{12} = 0,$$

with $\mu_{I_{12}} = 1.38\ \text{d}^{-1}$ [22], $K_{I_{12}} = 10^{-10}\ \text{g/cm}^3$, and $K_D = 4 \times 10^{-5}\ \text{g/cm}^3$, we get

$$\lambda_{I_{12}D} = 3.45 \times 10^{-6}\ \text{d}^{-1}.$$

Eq. (12)**:** We use the steady state equation

$$\lambda_{PC}C - \mu_P P = 0,$$

where $\mu_P = 4.16\ \text{d}^{-1}$, $K_C = 0.4\ \text{g/cm}^3$, and $K_P = 2 \times 10^{-7}\ \text{g/cm}^3$ [49]. Hence,

$$\lambda_{PC} = 2.1 \times 10^{-6}\ \text{d}^{-1}.$$

3 Parameter Sensitivity Analysis

We performed sensitivity analysis with a group of parameters, mostly production rates, which were roughly estimated. The computations were done using Latin Hypercube Sampling/Partial Rank Correlation Coefficient (LHS/PRCC) with a Matlab package by [50, 51]. The range for the parameters in the sensitivity analysis were between ±50% of their baseline values in Tables 1–2, except for λ_C which was chosen between ±10% of the baseline in Table 1.

The parameter λ_C is clearly positively correlated, and so is the cancer-specific parameter $\lambda_{T_1T_r}$, since an increase in the $T_1 \to T_r$ transition is pro-cancer. The other cancer-specific parameter ζ_{T_β} is also positively correlated with the tumor volume as it should be, by the inhibiting role it plays in Eq. (2). The parameters $\lambda_{T_1I_{12}}$ and $\lambda_{T_rT_\beta}$ are positively correlated; indeed, as they increase, the blockade of T_8 is increased. On the other hand, μ_{T_8C}, the killing rate of cancer by T_8, is negatively correlated, and so are $\lambda_{T_8I_{12}}$ and λ_{DC}, since an increase in these parameters results in an increase in T_8 cells and, respectively, in increased activation of T_8 cells by dendritic cells. M_2 is clearly pro-cancer, and this is shown in the positive correlation of λ_{M_2}. But, interestingly, Figure 1 shows that also M_1 is pro-cancer, as λ_{M_1} is positively correlated. Since λ_{M_2} is more positively correlated with tumor volume than λ_{M_1}, we may conclude that M_2 is more pro-cancer than M_1, and this is confirmed by noting that the rate of $M_1 \to M_2$, $\lambda_{M_1M_2}$, is positively correlated with the tumor volume, while the rate of $M_2 \to M_1$, $\lambda_{M_2M_1}$, is negatively correlated. Since M_1 appears in the model equations only in connection with M_2, we may conclude that its anti-cancer role is in increasing the number of M_2 cells.

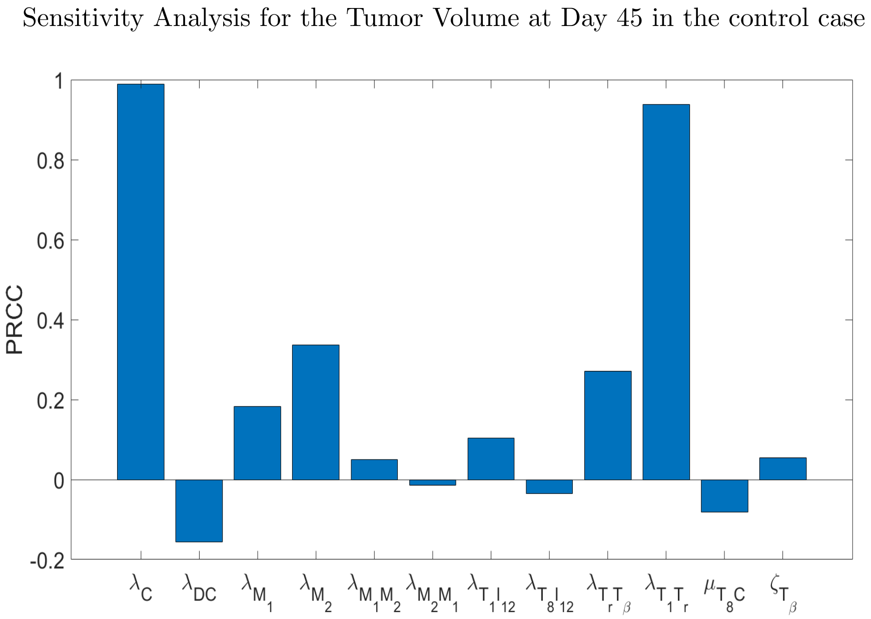

Fig 1. Parameter sensitivity analysis for the tumor volume; the p-value is < 0.01.

4 Numerical Method

We used the moving mesh method [52] to numerically solve the free boundary problem for the tumor proliferation model. To illustrate in this model, we take Eq. (2) as example and rewrite it in the following form:

$$\frac{\partial C(r,t)}{\partial t} = \delta_C \Delta C(r,t) - \nabla \cdot (\mathbf{u}C) + F, \tag{28}$$

where F represents the term in the right-hand side of Eq. (2). Let r_i^k and C_i^k denote numerical approximations of i-th grid point and $C(r_i^k, n\tau)$, respectively, where τ is the size of time-step. The discretization of Eq. (28) is derived by the fully implicit finite difference scheme:

$$\frac{C_i^{k+1} - C_i^k}{\tau} = \delta_C \left(C_{rr} + \frac{2}{r_i^k} C_r \right) - \left(\frac{2}{r_i^{k+1} u_i^{k+1} + u_r} \right) C_i^{k+1} - u_i^{k+1} C_r + F_i^{k+1},$$

where $C_r = \frac{h_{-1}^2 C_{i+1}^{k+1} - h_1^2 C_{i-1}^{k+1} - (h_1^2 - h_{-1}^2) C_i^{k+1}}{h_1(h_{-1}^2 - h_1 h_{-1})}$, $C_{rr} = 2\frac{h_{-1} C_{i+1}^{k+1} - h_1 C_{i-1}^{k+1} + (h_1 - h_{-1}) C_1^{k+1}}{h_1(h_1 h_{-1} - h_{-1}^2)}$,
$u_r = \frac{h_{-1}^2 u_{i+1}^{k+1} - h_1^2 u_{i+1}^{k+1} - (h_1^2 - h_{-1}^2) u_i^{k+1}}{h_1(h_{-1}^2 - h_1 h_{-1})}$, $h_{-1} = r_{i-1}^{k+1} - r_i^{k+1}$ and $h_1 = r_{i+1}^{k+1} - r_i^{k+1}$. The mesh moves by $r_i^{k+1} = r_i^k + u_i^{k+1}\tau$, where u_i^{k+1} is solved by the velocity equation.

In order to make the scheme stable, we take $\tau \le \frac{h^2}{4\delta_C}$, namely $\tau = 0.1\frac{h^2}{\delta_C}$, where $h = \min(h_{-1}, h_1)$.

References

1. Palucka J, Banchereau J. Cancer immunotherapy via dendritic cells. Nat Rev Cancer. 2012;12(4):265–277.

Table 1. Parameters for the model.

Parameters	Descriptions	Values	references
λ_C	proliferation rate of C	0.739 d^{-1}	[22]
λ_{M_1}	proliferation rate of M_1	1.35 d^{-1}	[43]
λ_{M_2}	proliferation rate of M_2	1.01 d^{-1}	[43]
C_M	carrying capacity for C	0.8 g/cm^3	[43,44]
D_0	source of D	2.75×10^{-4} g/cm^3	[25,53]est.
M_0	source of M	3.75×10^{-3} g/cm^3	[23,53]est.
T_{10}	source of T_1 from naive $CD4^+$ T cells	2.5×10^{-3} g/cm^3	[24,53]est.
T_{80}	source of T_8 from naive $CD8^+$ T cells	1.25×10^{-3} g/cm^3	[24,53]est.
$\hat{T}_1$	inflow of T_1 across tumor boundary	2.5×10^{-3} g/cm^3	[24]est.
$\hat{T}_8$	inflow of T_8 across tumor boundary	1.25×10^{-3} g/cm^3	[24]est.
μ_C	death rate of C	0.17 d^{-1}	[22]
μ_{M_1}	death rate of M_1	3.3×10^{-2} d^{-1}	[35]est.
μ_{M_2}	death rate of M_2	9.9×10^{-2} d^{-1}	[35]est.
μ_D	death rate of D	0.1 d^{-1}	[45]
μ_{T_1}	death rate of T_1	0.197 d^{-1}	[46–48]
μ_{T_8}	death rate of T_8	0.18 d^{-1}	[46–48]
μ_{T_r}	death rate of T_r	0.25 d^{-1}	[36]est.
μ_P	decay rate of P	4.16 d^{-1}	[39]est.
μ_{I_2}	decay rate of I_2	2.376 d^{-1}	[54]
$\mu_{I_{10}}$	decay rate of I_{10}	8.3178 d^{-1}	[22]
$\mu_{I_{12}}$	decay rate of I_{12}	1.38 d^{-1}	[22]
μ_{T_β}	decay rate of T_β	399.25 d^{-1}	[37,38]est.
μ_Q	dissociation rate of PD-1 and PD-L1	6×10^4 d^{-1}	[15]
μ_{A_1}	decay rate of A_1	4.62×10^{-2} d^{-1}	[22,55]
μ_{A_β}	decay rate of A_β	1.78 d^{-1}	[40]est.
$\mu_{P_D A_1}$	depletion rate of A_1 by P_D	1.2×10^7 $cm^3/g{\cdot}d$	[22]est.
$\mu_{T_\beta A_\beta}$	depletion rate of A_β by T_β	10^2 $cm^3/g{\cdot}d$	assumed
$\mu_{A_\beta T_\beta}$	depletion rate of T_β by A_β	10^5 $cm^3/g{\cdot}d$	assumed
δ_X	diffusion coefficient of cells	8.64×10^{-7} cm^2d^{-1}	[34]
δ_{I_2}	diffusion coefficient of IL-2	9.58×10^{-2} cm^2d^{-1}	[43]
δ_P	diffusion coefficient of CCL2	0.112 cm^2d^{-1}	[29]est.
$\delta_{I_{10}}$	diffusion coefficient of IL-10	9.11×10^{-2} cm^2d^{-1}	[29]est.
$\delta_{I_{12}}$	diffusion coefficient of IL-12	7.5×10^{-2} cm^2d^{-1}	[29]est.
δ_{T_β}	diffusion coefficient of TGF-β	14.8×10^{-2} cm^2d^{-1}	[31]est.
δ_{A_1}	diffusion coefficient of anti-PD-1	7.87×10^{-2} cm^2d^{-1}	[29,32]est.
δ_{A_β}	diffusion coefficient of anti-TGF-β	4.7×10^{-2} cm^2d^{-1}	[33]est.
ε_T	fraction of P_D expression in T_r	0.01	[22,42]est.
ε_C	fraction of P_L expression in C	0.01	[22,42]est.
ε_M	fraction of P_L expression in M_2	0.01	[22,42]est.
ρ_{P_L}	fraction mass of P_L in T_1 cell	5.22×10^{-7}	[22]est.
σ_0	rate of flux of T_8 at boundary	1 cm^{-1}	[44]
θ	average density of cells	0.6 g/cm^3	[22]
ζ_{T_β}	C-killing by T_8 inhibitory constant	$4 \times 10^5 - 4 \times 10^6$ cm^3/g	assumed

est.= this parameter was estimated in Section 2. $X \in \{C,\ D,\ M_1,\ M_2,\ T_1,\ T_8,\ T_r\}$.

Table 2. Parameters for the model (continued).

Parameters	Descriptions	Values	references
μ_{T_8C}	killing rate of C by T_8	1347.02 cm^3/g·d	est.
λ_{DC}	activation rate of DCs by C	0.053 d^{-1}	est.
$\lambda_{T_1I_2}$	activation rate of T_1 by IL-2	0.25 d^{-1}	[43]
$\lambda_{T_8I_2}$	activation rate of T_8 by IL-2	0.25 d^{-1}	[43]
$\lambda_{T_rI_2}$	activation rate of T_r by IL-2	0.25 d^{-1}	[43]
$\lambda_{T_1I_{12}}$	activation rate of T_1 by IL-12	27.26 d^{-1}	[43]
$\lambda_{T_8I_{12}}$	activation rate of T_8 by IL-12	0.486 d^{-1}	est.
λ_{PC}	production rate of P by C	2.1×10^{-6} d^{-1}	est.
$\lambda_{I_2T_1}$	production rate of IL-2 by T_1	1.15×10^{-4} d^{-1}	[53, 56]
$\lambda_{I_{10}C}$	production rate of IL-10 by C	1.73×10^{-8} d^{-1}	est.
$\lambda_{I_{10}M_2}$	production rate of IL-10 by M_2	1.1×10^{-5} d^{-1}	est.
$\lambda_{I_{12}D}$	production rate of IL-12 by DCs	3.45×10^{-6} d^{-1}	est.
$\lambda_{M_1M_2}$	rate of $M_1\to M_2$ transition	0.69 d^{-1}	[53, 57]
$\lambda_{M_2M_1}$	rate of $M_2\to M_1$ transition	3.61 d^{-1}	[53]
$\lambda_{T_rT_\beta}$	T_β-enhanced activation rate of T_r	1.5 d^{-1}	[43]
$\lambda_{T_1T_r}$	rate of $T_1\to T_r$ transition	6.24–1×10^3 d^{-1}	assumed
$\lambda_{T_\beta C}$	rate of secretion of T_β by C	5.39×10^{-11} d^{-1}	[43]
$\lambda_{T_\beta M_2}$	rate of secretion of T_β by M_2	1.35×10^{-9} d^{-1}	[43]
$\lambda_{T_\beta T_r}$	rate of secretion of T_β by T_r	2.16×10^{-7} d^{-1}	[43]
K_P	average of P	2×10^{-7} g/cm^3	[49]
K_Q	average level of Q	4.86×10^{-20} g^2/cm^6	[13, 14]est.
K_C	average level of C	0.4 g/cm^3	[43, 44]
$K_{I_{12}}$	average level of IL-12	10^{-10} g/cm^3	[20]est.
$K_{I_{10}}$	average level of IL-10	5×10^{-9} g/cm^3	[19]est.
$\hat{K}_{TI_{10}}$	inhibition of $T_1,\ T_8$ by IL-10	5×10^{-9} g/cm^3	[19]est.
K_{T_β}	average level of T_β	7.2×10^{-9} g/cm^3	[17, 21]est.
$\hat{K}_{TT_r}$	inhibition of $T_1,\ T_8$ by T_r	2.5×10^{-4} /cm^3	[43]
$\hat{K}_{TQ}$	inhibition of T_8 by P_D-P_L	4.86×10^{-20} g^2/cm^6	[13, 14]est.
K_D	average level of D	4×10^{-5} g/cm^3	[16]est.
K_{M_1}	average level of M_1	10^{-4} g/cm^3	[43]
K_{M_2}	average level of M_2	3.2×10^{-3} g/cm^3	[43]
K_{T_1}	average level of T_1	2×10^{-3} g/cm^3	[43]
K_{T_8}	average level of T_8	5×10^{-4} g/cm^3	[18]est.
χ_P	chemoattraction coefficient of by P	10 cm^5/g·d	[43]

est.= this parameter was estimated in Section 2.

2. Saenz R, Futalan D, Leutenez L, Eekhout F, Fecteau JF, Sundelius S, et al. Tlr4-dependent activation of dendritic cells by an hmgb1-derived peptide adjuvant. J Transl Med. 2014;12(211):1–11.

3. Ma Y, Shurin GV, Peiyuan Z, Shurin MR. Dendritic cells in the cancer microenvironment. J Cancer. 2013;4(1):36–44.

4. Perrot CY, Javelaud D, Mauviel A. Insights into the transforming growth factor-beta signaling pathway in cutaneous melanoma. Ann Dermatol. 2013;25(2):135–144.

5. Janco JMT, Lamichhane P, Karyampudi L, Knutson KL. Tumor-infiltrating dendritic cells in cancer pathogenesis. J Immunol. 2015;194(7):2985–2991.

6. Kawakami Y, Yaguchi T, Sumimoto H, Kudo-Saito C, Iwata-Kajihara T, Nakamura S, et al. Improvement of cancer immunotherapy by combining molecular targeted therapy. Front Oncol. 2013;3(136):1–7.

7. Ilieva KM, Correa I, Josephs DH, Karagiannis P, Egbuniwe IU, Cafferkey MJ, et al. Effects of BRAF mutations and BRAF inhibition on immune responses to melanoma. Mol Cancer Ther. 2014;13(12):2769–2783.

8. Oelkrug C, Ramage JM. Enhancement of t cell recruitment and infiltration into tumours. Clin Exp Immunol. 2014;178(1):1–8.

9. Umansky V, Blattner C, Gebhardt C, Utikal J. The Role of Myeloid-Derived Suppressor Cells (MDSC) in Cancer Progression. Vaccines (Basel). 2016;4(36):1–16.

10. Condamine T, Gabrilovich DI. Molecular mechanisms regulating myeloid-derived suppressor cell differentiation and function. Trends Immunol. 2011;32(1):19–25.

11. Cantelli G, Crosas-Molist E, Georgouli M, Sanz-Moreno V. TGFb-induced transcription in cancer. Semin Cancer Biol. 2016;42:60–69.

12. Whiteside TL. The role of regulatory t cells in cancer immunology. Immunotargets Ther. 2015;4:159–171.

13. Shi L, Chen S, Yang L, Li Y. The role of PD-1 and PD-L1 in T cell immune suppression in patients with hematological malignancies. J Hematol Oncol. 2013;6(74):10–11861768722674.

14. Muppidi MR, George S. Immune checkpoint inhibitors in renal cell carcinoma. J Targeted Ther Cancer. 2015;4:47–52.

15. Maute RL, Gordon SR, Mayer AT, McCracken MN, Natarajan A, Ring NG, et al. Engineering high-affinity PD-1 variants for optimized immunotherapy and immuno-PET imaging. Proc Natl Acad Sci USA. 2015;112(47):E6506–14.

16. Vescovi R, Monti M, Moratto D, Paolini L, Consoli F, Benerini L, et al. Collapse of the Plasmacytoid Dendritic Cell Compartment in Advanced Cutaneous Melanomas by Components of the Tumor Cell Secretome. Cancer Immunol Res. 2018;7(1):12–28.

17. Krombach F, Münzing S, Allmeling AM, Gerlach JT, Behr J, M MD. Cell size of alveolar macrophages: an interspecies comparison. Environ Health Perspect. 1997;105(5):1261–1265. doi:10.1289/ehp.97105s51261.

18. Lisiero DN, Soto H, Liau LM, Prins RM. Enhanced sensitivity of IL-2 signaling regulates the clinical responsiveness of IL-12-primed CD8^{+} T cells in a melanoma model. J Immunol. 2011;186:5068–5077.

19. Kruger-Krasagakes S, Krasagakis K, Garbe C, Schmitt E, Huls C, Blankenstein T, et al. Expression of interleukin 10 in human melanoma. Br J Cancer. 1994;70:1182–1185.

20. Jafarzadeh A, Minaee K, Farsinejad A, Nemati M, Khosravimashizi A, Daneshvar H, et al. Evaluation of the circulating levels of IL-12 and IL-33 in patients with breast cancer: influences of the tumor stages and cytokine gene polymorphisms. Iran J Basic Med Sci. 2015;18(12):1189–1198.

21. Berking C, Takemoto R, Schaider H, Showe L, Satyamoorthy K, Robbins P, et al. Transforming Growth Factor-β1 Increases Survival of Human Melanoma through Stroma Remodeling. Cancer Res. 2001;61:8306–8316.

22. Lai X, Friedman A. Combination therapy for melanoma with BRAF/MEK inhibitor and immune checkpoint inhibitor: a mathematical model. BMC Systems Biology. 2017;11(1):1–18.

23. Lee SH, Starkey PM, Gordon S. Quantitaive Analysis of Total Macrophage Content in Adult Mouse Tissues: Immunochemical Studies with Monoclonal Antibody F4/80. J Exp Med. 1985;161:475–489.

24. Tsukahara A, Seki S, Iiai T, Moroda T, Watanabe H, Suzuki S, et al. MOuse Liver T Cells: Their Change With Aging and in Comparison With Peripheral T Cells. Hepatology. 1997;26:301–309.

25. Jomantaite I, Dikopoulos N, Kroger A, Leithauser F, Hauser H, Schirmbeck R, et al. Hepatic dendritic cell subsets in the mouse. Eur J Immunol. 2004;34(2):355–365.

26. Simpson LO. Spleen and Liver Weights Change in NZB Mouse with Haemolytic Anaemia. Laboratory Animals. 1975;9:261–273.

27. Yin M, Bradford BU, Wheeler MD, Uesugi T, Froh M, Goyert SM, et al. Reduced Early Alcohol-Induced Liver Injury in CD14-Deficient Mice. J Immunol. 2001;166:4737–4742.

28. Young ME. Estimation of diffusion coefficients of proteins. Biotech Bioeng. 1980;XXII:947–955.

29. Hornbeck PV, Zhang B, Murray B, Kornhauser JM, Latham V, Skrzypek E. PhosphoSitePlus, 2014: mutations, PTMs and recalibrations. Nucleic Acids Research. 2015;43:D512–D520.

30. Liao KL, Bai XF, Friedman A. Mathematical modeling of interleukin-27 induction of anti-tumor T cells response. PLoS ONE. 2014;9(3).

31. National Center for Biotechnology Information. PubChem Compound Summary for CID 56842206, TGF-beta. PubChem, https://pubchemncbinlmnihgov/compound/TGF-beta. Accessed January 24, 2021;.

32. Abcam. Anti-PD1 antibody (ab89828). http://wwwabcamcom/pd1-antibody-ab89828html;.

33. Abcam. Anti-TGF-beta 1 antibody (ab92486). https://wwwabcamcom/tgf-beta-1-antibody-ab92486html?productWallTab=ShowAll;.

34. Kim Y, Lawler S, Nowicki MO, Chiocca EA, Friedman A. A mathematical model for pattern formation of glioma cells outside the tumor spheroid core. J Theor Biol. 2009;260(3):359–371.

35. Italiani P, Boraschi D. From Monocytes to M1/M2 Macrophages: Phenotypical vs. Functional Differentiation. Front Immunol. 2014;5(514):1–22.

36. Furlan SN, Singh K, Lopez C, Tkachev V, Hunt DJ, Hibbard J, et al. IL-2 enhances ex vivo–expanded regulatory T-cell persistence after adoptive transfer. Blood Adv. 2020;4(8):1594–1605.

37. Prieto M, Rivas JV, López Novoa JM, Pérez-Barriocanal F. TGF-beta: synthesis and mechanism of action. Nefrologia. 2002;22(2):135–143.

38. Tirado-Rodriguez B, Ortega E, Segura-Medina P, Huerta-Yepez S. TGF-β: An Important Mediator of Allergic Disease and a Molecule with Dual Activity in Cancer Development. J Immunol Res. 2014;2014(318481):1–15. doi:10.1155/2014/318481.

39. Cheng J, Diaz Encarnacion MM, Warner GM, Gray CE, Nath KA, Grande JP. TGF-β1 stimulates monocyte chemoattractant protein-1 expression in mesangial cells through a phosphodiesterase isoenzyme 4-dependent process. Am J Physiol Cell Physiol. 2005;289(4):C959–70. doi:10.1152/ajpcell.00153.2005.

40. Cassidy KC, Gueorguieva I, Miles C, Rehmel J, Yi P, Ehlhardt WJ. Disposition and metabolism of [^{14}C]-galunisertib, a TGF-βRI kinase/ALK5 inhibitor, following oral administration in healthy subjects and mechanistic prediction of the effect of itraconazole on galunisertib pharmacokinetics. Xenobiotica. 2018;48(4):382–399. doi:10.1080/00498254.2017.1323137.

41. Toossi Z, Hirsch CS, Hamilton BD, Knuth CK, Friedlander MA, Rich EA. Decreased production of TGF-beta 1 by human alveolar macrophages compared with blood monocytes. J Immunol. 1996;156(9):3461–3468.

42. Butte MJ, Pena-Cruz V, Kim MJ, Freeman GJ, Sharpe AH. Interaction of human PD-L1 and B7-1. Mol Immunol. 2008;45(13):3567–3572.

43. Lai X, Stiff A, Duggan M, Wesolowski R, Carson III WE, Friedman A. Modeling combination therapy for breast cancer with BET and immune checkpoint inhibitors. PNAS. 2018;115(21):5534–5539.

44. Friedman A, Hao W. The Role of Exosomes in Pancreatic Cancer Microenvironment. Bull Math Biol. 2018;80(5):1111–1133.

45. Hao W, Crouser ED, Friedman A. Mathematical Model of Sarcoidosis. PNAS. 2014;111(45):16065–16070.

46. Crouser ED, Knox KS, Julian MW, Shao G, Abraham S, Liyanarachchi S, et al. Gene expression profiling identifies MMP-12 and ADAMDEC1 as potential pathogenic mediators of pulmonary sarcoidosis. Am J Respir Crit Care Med. 2009;179(10):929–938.

47. Hengel RL, Jones BM, Kennedy MS, Hubbard MR, McDougal JS. Markers of Lymphocyte Homing Distinguish CD4 T Cell Subsets That Turn Over in Response to HIV-1 Infection in Humans. J Immunol. 1999;163:3539–3548.

48. Mulligan JK, Rosenzweig SA, Young MRI. Tumor secretion of vegf induces endothelial cells to suppress t cell functions through the production of pge2. J Immunother (Hagerstown, MD: 1997). 2010;33(2):126–135.

49. Hao W, Schlesinger LS, Friedman A. Modeling Granuloma in Response to Infection in the Lung. PLoS ONE. 2016;11(3):1–26.

50. Kirschner DE. Uncertainty and sensitivity functions and implementation. http://malthus.micro.med.umich.edu/lab/usadata/: University of Michigan; 2007–2008.

51. Marino S, Hogue IB, Ray CJ, Kirschner DE. A methodology for performing global uncertainty and sensitivity analysis in systems biology. J Theor Biol. 2008;254:178–196.

52. D'Acunto B. Computational Methods for PDE in Mechanics, Series on Advances in Mathematics for Applied Sciences. World Scientific. 2004;67.

53. Siewe N, Yakubu AA, Satoskar AR, Friedman A. Immune Response to Infection by Leishmania: A Mathematical Model. Mathematical Biosciences. 2016;276:28–43.

54. Donohue JH, Rosenberg SA. The fate of interleukin-2 after *in vivo* administration. J Immunol. 1983;130(5):2203–2208.

55. Brahmer JR, Drake CG, Wollner I, Powderly JD, Picus J, Sharfman WH, et al. Phase I study of single-agent anti-programmed death-1 (MDX-1106) in refractory solid tumors: safety, clinical activity, pharmacodynamics, and immunologic correlates. J Clin Oncol. 2010;28(19):3167–3175.

56. Balestrino M. Cytokine Imbalances in Multiple Sclerosis: A Computer Simulation. M Eng Projects. 2009;.

57. Oliver JC, *et al* . Cytokines kinetics in an in vitro whole blood model following an endotoxin challenge. Lymphokine Cytokine Res. 1993;12(2):115–120.
